# Treating Spin–Orbit Coupling and Spin–Spin Coupling in the Framework of the Iterative Configuration Expansion Selected CI

**DOI:** 10.1021/acs.jctc.5c00463

**Published:** 2025-06-23

**Authors:** Lucas Lang, Vijay Gopal Chilkuri, Frank Neese

**Affiliations:** 28314 Max-Planck-Institut für Kohlenforschung, Kaiser-Wilhelm-Platz 1, 45470 Mülheim an der Ruhr, Germany

## Abstract

Spin-adapted configuration state functions (CSFs) provide a compact many-electron basis for open-shell molecules. This basis is employed in one flavor of the recently introduced iterative configuration expansion (ICE) selected CI method. In this work, we implemented spin-dependent operators like spin–orbit coupling and direct spin–spin coupling for use in quasidegenerate perturbation theory on top of nonrelativistic/scalar-relativistic ICE wave functions. At the core of the new implementation are matrix elements of spin tensor excitation operators between CSFs, which are evaluated as products of orbital-specific factors. Two applications, the electron paramagnetic resonance g-factors of a Mo^III^-based catalytic intermediate and the zero-field splitting in dioxygen, illustrate the capabilities of the new method.

## Graphical abstract

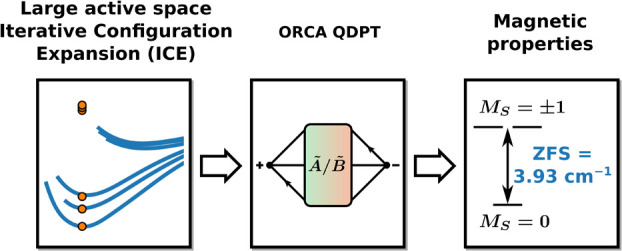

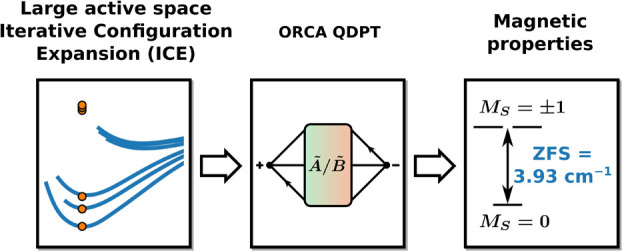

## 1 Introduction

In recent years, there has been increased interest in methods for approximately solving very large full configuration interaction (FCI) or complete active space (CAS) problems. Examples for methods in this area are the density matrix renormalization group (DMRG),^1–7^ the full configuration interaction quantum Monte Carlo (FCIQMC),^8–10^ the many-body expanded FCI,^11–15^ as well as various flavors of selected CI, like the (semistochastic) heat-bath CI,^16–18^ the adaptive sampling selected CI,^19,20^ the iterative CI,^21,22^ as well as our own iterative configuration expansion (ICE) selected CI method,^23,24^ which is based on the CIPSI method.^25^

In most cases, these methods were initially used together with nonrelativistic or scalar-relativistic Hamiltonians, and thus could not describe effects like zero-field splitting (ZFS) that depend on spin-dependent operators such as spin–orbit coupling (SOC) and dipolar spin–spin coupling (SSC). While extensions to fully relativistic Hamiltonians were reported,^26,27^ they come with a significant increase in computational cost.

More economical are methods that use scalar orbitals and consider the spin-dependent operators only at the many-electron level. Examples are one-step approaches like the spin–orbit heat-bath CI of Mussard and Sharma^28^ and the SOiCI method of Liu and co-workers,^29^ as well as two-step approaches like the DMRG/QDPT method by Roemelt,^30^ DMRG state interaction methods by Knecht at al.^31,32^ and Sayfutyarova and Chan,^33^ and the iCISO method of Liu and co-workers.^29^

One variant of the ICE method uses configuration state functions (CSFs) as many-electron basis functions, which hold great promise for an efficient description of molecules with a large number of unpaired electrons, such as magnetically coupled polynuclear transition metal clusters. Li Manni and co-workers^34–37^ as well as two of us^38^ have shown in recent years that using a CSF basis can lead to very compact representations of the wave functions of such systems. In this work, we implement triplet one-electron operators (such as an effective one-electron SOC operator) and quintet two-electron operators (i.e., the SSC operator) in the framework of the CSF-based ICE method. We make heavy use of diagrammatic techniques for angular momentum coupling, which were already pioneered in the early days of the development of the (graphical) unitary group approach ((G)­UGA)^39^ and earlier work by Gouyet^40–42^ and Drake and Schlesinger.^43^ Such graphical techniques were also used in the development of the spin-independent ICE method.^23,24^ We provide an alternative route toward these diagrams that allows us to significantly simplify the treatment of SSC compared to previously published work. Finally, we describe the implementation of these spin-dependent operators at the level of quasi-degenerate perturbation theory (QDPT)^44^ on top of nonrelativistic/scalar-relativistic ICE wave functions and provide two illustrative applications.

## 2 Theory

### 2.1 Spin-Dependent Operators Considered in This Work

In this work, we consider matrix elements of triplet one-electron operators, such as the spin–orbit mean field (SOMF) operator,^45,46^ and matrix elements of quintet two-electron operators, for ICE states |Ψ_*J*_^*SM*^⟩, which are linear combinations of CSFs |Φ_*L*_^*SM*^⟩,

$$\left|\Psi_{J}^{SM}\rangle =\underset{L}{\sum }|\Phi_{L}^{SM}\right\rangle C_{LJ}^{S}$$ 1

In the following, we use Hartree atomic units and *g*_e_ and α are the free-electron *g*-value and the fine-structure constant, respectively.

#### 2.1.1 Triplet Operators

An arbitrary one-electron triplet operator, such as the SOMF operator, can be decomposed as

$$A_{T}=\underset{m}{\sum }A_{T}^{\left(m\right)}\left(m\right)$$ 2

In the notation *A*_*T*_^(*m*)^(*m*′), *m* denotes the *component* of the spin tensor operator, whereas the label *m*′ distinguishes *different* spin tensor operators. Using the Wigner–Eckart theorem, one can write

$$\left\langle \Psi_{I}^{S^{\prime }M^{\prime }}\right|A_{T}|\Psi_{J}^{SM}\rangle =\underset{m}{\sum }\left(\begin{array}{cc} S & 1\\ M & m \end{array}|\begin{array}{c} S^{\prime }\\ M^{\prime } \end{array}\right)\left\langle \Psi_{I}^{S^{\prime }}∥A_{T}\left(m\right)∥\Psi_{J}^{S}\right\rangle =\left(\begin{array}{cc} S & 1\\ M & M^{\prime }-M \end{array}|\begin{array}{c} S\\ M^{\prime } \end{array}\right)\left\langle \Psi_{I}^{S^{\prime }}∥A_{T}\left(M^{\prime }-M\right)∥\Psi_{J}^{S}\right\rangle$$ 3

In the second step, we made use of the requirement *M* + *m* = *M*′ needed for nonzero Clebsch–Gordan coefficients (CGCs). We can also first expand the states into CSFs and apply the Wigner–Eckart theorem afterward,

$$\left\langle \Psi_{I}^{S^{\prime }M^{\prime }}\right|A_{T}|\Psi_{J}^{SM}\rangle =\underset{KL}{\sum }C_{KI}^{S^{\prime }}C_{LJ}^{S}\left\langle \Phi_{K}^{S^{\prime }M^{\prime }}\right|A_{T}|\Phi_{L}^{SM}\rangle =\underset{KL}{\sum }C_{KI}^{S^{\prime }}C_{LJ}^{S}\left(\begin{array}{cc} S & 1\\ M & M^{\prime }-M \end{array}|\begin{array}{c} S\\ M^{\prime } \end{array}\right)\left\langle \Phi_{K}^{S^{\prime }}∥A_{T}\left(M^{\prime }-M\right)∥\Phi_{L}^{S}\right\rangle$$ 4

Comparison of eqs 3 and 4 shows that

$$\left\langle \Psi_{I}^{S^{\prime }}∥A_{T}\left(m^{\prime }\right)∥\Psi_{J}^{S}\right\rangle =\underset{KL}{\sum }C_{KI}^{S^{\prime }}C_{LJ}^{S}\left\langle \Phi_{K}^{S^{\prime }}∥A_{T}\left(m^{\prime }\right)∥\Phi_{L}^{S}\right\rangle$$ 5

The components of the general triplet operator can be written^47^

$$A_{T}^{\left(m\right)}\left(m^{\prime }\right)=\underset{pq}{\sum }a_{pq}\left(m^{\prime }\right)s_{pq}^{\left(m\right)}$$ 6

where the *a*_*pq*_(*m*′) are integrals and *s*_*pq*_^(*m*)^ is the triplet single-excitation operator with magnetic quantum number *m*. This leads to

$$\left\langle \Phi_{K}^{S^{\prime }}∥A_{T}\left(m^{\prime }\right)∥\Phi_{L}^{S}\right\rangle =\underset{pq}{\sum }a_{pq}\left(m^{\prime }\right)\left\langle \Phi_{K}^{S^{\prime }}∥s_{pq}∥\Phi_{L}^{S}\right\rangle$$ 7

One can now see that the calculation of matrix elements of triplet one-electron operators is reduced to the calculation of the coupling coefficient reduced matrix elements (RMEs) ⟨Φ_*K*_^*S* ^′^^∥*s*_*pq*_∥Φ_*L*_^*S*^⟩.

#### 2.1.2 The Direct Spin–Spin Coupling Operator

The direct spin–spin coupling operator is a part of the Breit–Pauli Hamiltonian that can be interpreted as the magnetic dipole–dipole interaction between electron spins. It can be written as

$$H_{\mathrm{SSC}}=-\frac{g_{e}^{2}\alpha^{2}}{4}\underset{i<j}{\sum }\frac{3\left(r_{ij}\cdot s_{i}\right)\left(r_{ij}\cdot s_{j}\right)-r_{ij}^{2}s_{i}\cdot s_{j}}{r_{ij}^{5}}$$ 8

This is a pure quintet two-electron operator. It can be decomposed into a sum of components

$$H_{\mathrm{SSC}}=\underset{m=0,\pm 1,\pm 2}{\sum }H_{\mathrm{SSC}}^{\left(m\right)}\left(m\right)$$ 9

which, in second quantization, are given by

$$H_{\mathrm{SSC}}^{\left(m\right)}\left(m^{\prime }\right)=\frac{{\left(-1\right)}^{m^{\prime }}}{2}\underset{pqrs}{\sum }d_{pqrs}^{\left(-m^{\prime }\right)}S_{pqrs}^{\left(m\right)}$$ 10

Here, the dipolar two-electron integrals are defined as

$$d_{pqrs}^{\left(-m^{\prime }\right)}=\left\langle pr\right|d_{12}^{\left(-m^{\prime }\right)}|qs\rangle$$ 11

$$d_{12}^{\left(kl\right)}=-\frac{g_{e}^{2}\alpha^{2}}{4}\frac{3r_{12}^{\left(k\right)}r_{12}^{\left(l\right)}-\delta_{kl}r_{12}^{2}}{r_{12}^{5}}$$ 12

We use Mulliken ordering of orbital labels (as is common for the *e*_*pqrs*_ singlet double excitation operator), i.e., the quintet double excitation operators *S*_*pqrs*_^(*m*)^ (defined in Section 2.2.2 below) annihilate electrons in orbitals *q* and *s* and create electrons in orbitals *p* and *r*. There is the permutational (anti)­symmetry^47,48^

$$S_{pqrs}^{\left(m\right)}=-S_{rqps}^{\left(m\right)}=-S_{psrq}^{\left(m\right)}=S_{rspq}^{\left(m\right)}$$ 13

Using these identities together with *d*_*pqrs*_^(−*m* ^′^)^ = *d*_*rspq*_^(−*m* ^′^)^ leads to

$$H_{\mathrm{SSC}}^{\left(m\right)}\left(m^{\prime }\right)={\left(-1\right)}^{m^{\prime }}\underset{\begin{array}{c} p<r\\ q<s \end{array}}{\sum }\left[d_{pqrs}^{\left(-m^{\prime }\right)}-d_{psrq}^{\left(-m^{\prime }\right)}\right]S_{pqrs}^{\left(m\right)}$$ 14

i.e., we can restrict ourselves to deriving equations for matrix elements of operators *S*_*pqrs*_^(*m*)^ where the orbitals involved in the excitation satisfy *p* < *r* and *q* < *s*.

In complete analogy to the triplet operator case, the Wigner–Eckart theorem can be used to write

$$\left\langle \Psi_{I}^{S^{\prime }M^{\prime }}\right|H_{\mathrm{SSC}}|\Psi_{J}^{SM}\rangle =\left(\begin{array}{cc} S & 2\\ M & M^{\prime }-M \end{array}|\begin{array}{c} S^{\prime }\\ M^{\prime } \end{array}\right)\left\langle \Psi_{I}^{S^{\prime }}∥H_{\mathrm{SSC}}\left(M^{\prime }-M\right)∥\Psi_{J}^{S}\right\rangle$$ 15

with

$$\left\langle \Psi_{I}^{S^{\prime }}∥H_{\mathrm{SSC}}\left(m^{\prime }\right)∥\Psi_{J}^{S}\right\rangle =\underset{KL}{\sum }C_{KI}^{S^{\prime }}C_{LJ}^{S}\underset{\begin{array}{c} p<r\\ q<s \end{array}}{\sum }{\left(-1\right)}^{m^{\prime }}\left[d_{pqrs}^{\left(-m^{\prime }\right)}-d_{psrq}^{\left(-m^{\prime }\right)}\right]\left\langle \Phi_{K}^{S^{\prime }}∥S_{pqrs}∥\Phi_{L}^{S}\right\rangle$$ 16

Again, the task is essentially reduced to the calculation of coupling coefficient RMEs ⟨Φ_*K*_^*S* ^′^^∥*S*_*pqrs*_∥Φ_*L*_^*S*^⟩.

### 2.2 CSF Matrix Elements of Excitation Operators in Terms of Pure Spin Functions

Using a first-quantization approach, Wormer and Paldus derived equations for the matrix elements of spin-traced excitation operators *E*_*pq*_ and *e*_*pqrs*_ between CSFs in terms of matrix elements of pure spin functions.^49^ In this section, we derive a generalization of this result to arbitrary single- and double-excitation operators (in particular in order to obtain equations for the coupling coefficient RMEs ⟨Φ_*K*_^*S* ^′^^∥*s*_*pq*_∥Φ_*L*_^*S*^⟩ and ⟨Φ_*K*_^*S* ^′^^∥*S*_*pqrs*_∥Φ_*L*_^*S*^⟩). This enables the treatment of arbitrary one- and two-electron operators in a two-component relativistic picture.

Following Wormer and Paldus,^49^ an *N*-electron CSF is defined as

$$|\Phi_{J}^{SM}\rangle =N_{J}A\left(|\Phi_{J}\rangle |X_{J}^{SM}\rangle \right)$$ 17

where *N*_*J*_ is a normalization constant and

$$A=\frac{1}{N!}\underset{\sigma \in S_{N}}{\sum }\mathrm{sgn}\left(\sigma \right)P_{\sigma }$$ 18

is the antisymmetrizer, where *S*_*N*_ is the symmetric group, sgn­(σ) is the parity (±1) of the permutation σ, and *P*_σ_ is the representation of the permutation on *N*-electron space. |Φ*_J_* ⟩ is a simple Hartree product of orthonormal spatial orbitals,

$$|\Phi_{J}\rangle =\psi_{p_{1}}\left(r_{1}\right)\cdot \cdot \cdot \psi_{p_{N}}\left(r_{N}\right)$$ 19

where the orbitals are assumed to be in canonical order, i.e. *p*_*i*_ ≤ *p*_*j*_ for *i* < *j*, and |*X*_*J*_^*SM*^⟩ is an *N*-electron pure spin function. Each spatial orbital can occur up to twice in |Φ*_J_* ⟩, which then corresponds to a doubly occupied molecular orbital (DOMO). The invariance group S(J) is defined as the subgroup of *S*_*N*_ that leaves the orbital product |Φ*_J_* ⟩ invariant. The elements of S(J) are all possible combinations of transpositions between DOMO pairs. Therefore, its order is $\left|S\left(J\right)\right|=2^{N_{\mathrm{DOMO}}\left(J\right)}$ , where *N*_DOMO_(*J*) is the number of DOMOs in |Φ*_J_* ⟩). In this work, we assume that the |*X*_*J*_^*SM*^⟩ are “geminally antisymmetric”,^49^ which means that they are antisymmetric under permutations from S(J) . Expressed as an equation, geminal antisymmetry means that

$$P_{\tau }|X_{J}^{SM}\rangle =\mathrm{sgn}(\tau )|X_{J}^{SM}\rangle ,\mathrm{for}\;\tau \in S(J)$$ 20

If the geminally antisymmetric spin function is normalized to one, ⟨*X*_*J*_^*SM*^ | *X*_*J*_^*SM*^⟩ = 1, the normalization constant in eq 17 is given by $N_{J}=\sqrt{N!}{\left(\frac{1}{\sqrt{2}}\right)}^{N_{\mathrm{DOMO}}\left(J\right)}$ .^49^ Examples for geminally antisymmetric spin functions are Rumer spin functions and Yamanouchi-Kotani (YK)^50–52^ spin functions. Note that geminal antisymmetry is not a restriction: *Any* CSF (a simultaneous eigenfunction of **S** ^2^, *S*_*z*_, and the orbital occupation number operators *N*_*p*_ = *E*_*pp*_) can be written in the form of eq 17 with a geminally antisymmetric spin function; see Appendix 1.1 for a proof.

#### 2.2.1 One-Electron Operators

Within the four-dimensional space of operators acting on the two-dimensional Hilbert space of a single spin 1/2, the identity operator together with the three irreducible spin operators *s* ^*m*^ form a complete basis. Let *s* ^(*k*,*m*)^ be one of these operators, with *k* denoting the total spin of the operator. Then one can define a corresponding single-excitation operator via

$$s_{pq}^{\left(k,m\right)}=\overset{N}{\underset{i=1}{\sum }}|\psi_{p}\left(r_{i}\right)\rangle \left\langle \psi_{q}\left(r_{i}\right)\right|s_{i}^{\left(k,m\right)}$$ 21

The analogous Fock space operator is

$$s_{pq}^{\left(k,m\right)}=\underset{\sigma ,\lambda \in \left\{\alpha ,\beta \right\}}{\sum }\left\langle \sigma \right|s^{\left(k,m\right)}|\lambda \rangle a_{p\sigma }^{†}a_{q\lambda }$$ 22

Note that the operators defined in eqs 21 and 22 are not identical. The first one acts in the space of all (also nonantisymmetric) wave functions with definite electron number *N*, whereas the latter acts in the space of antisymmetric wave functions of arbitrary electron number (projected on a finite basis). However, the restrictions of these operators to the intersection of their domains, the space of antisymmetric *N*-electron wave functions (FCI space), is identical. When working within this space, eqs 21 and 22 can be used interchangeably. When choosing *k* = 0, which means *s* ^(*k*,*m*)^ = 1, eqs 21 and 22 define the singlet single-excitation operator *E*_*pq*_, while for *k* = 1 they define the triplet single-excitation operators *s*_*pq*_^*m*^. Any one-electron operator can be written in terms of integrals and these four single-excitation operators.

Following Wormer and Paldus, we use the version of the single-excitation operator that acts on *N*-electron space (eq 21). Let |Φ_*J*_^*SM*^⟩ be a CSF that has orbital *q* occupied at least once. Since eq 21 is a usual one-electron operator, it commutes with permutation operators and in particular with the antisymmetrizer. Therefore, one can directly act with it on the product of spatial and spin function,

$$s_{pq}^{\left(k,m\right)}\left(|\Phi_{J}\rangle |X_{J}^{SM}\rangle \right)=\underset{\tau \in S_{q}\left(J\right)}{\sum }\left(P_{\tau }|\Phi_{J}^{q\to p}\rangle \right)s_{\tau \left(i_{q}\right)}^{\left(k,m\right)}\left|X_{J}^{SM}\right\rangle =\underset{\tau \in S_{q}\left(J\right)}{\sum }\mathrm{sgn}\left(\tau \right)P_{\tau }\left(|\Phi_{J}^{q\to p}\rangle s_{i_{q}}^{\left(k,m\right)}|X_{J}^{SM}\rangle \right)$$ 23

Here, *i*_*q*_ is an electron position at which orbital *q* occurs in |Φ*_J_* ⟩ and the Hartree product |Φ_*J*_^*q* → *p*^⟩ is obtained from |Φ*_J_* ⟩ by replacing orbital *q* in position *i*_*q*_ by orbital *p*. Note that it is irrelevant which of the (up to two) positions of orbital *q* is chosen as *i*_*q*_. $S_{q}\left(J\right)$ is a new object introduced in the context of the present derivation. We call it the “reduced” invariance group of |Φ*_J_* ⟩ for orbital *q*. This group contains either only the identity (if *q* is a SOMO), or the identity and the transposition of the two positions where *q* occurs (if *q* is a DOMO). Hence, eq 23 is a sum over either one or two terms. In the second step of eq 23 we used that

$$P_{\sigma }o_{i}=o_{\sigma \left(i\right)}P_{\sigma }$$ 24

for an arbitrary permutation σ and operator *o*_*i*_ acting on the *i*th electron. We also used that τ^–1^ = τ for a permutation $\tau \in S_{q}\left(J\right)$ , and that the spin function is geminally antisymmetric (eq 20).

Now we assume that |Φ_*I*_^*S* ^′^ *M* ^′^^⟩ is another CSF defined according to eq 17 where the Hartree product |Φ*_I_* ⟩ contains exactly the same orbitals as |Φ_*J*_^*q* → *p*^⟩ but in canonical order. Note that this means that |Φ_*J*_^*SM*^⟩ and |Φ_*I*_^*S* ^′^ *M* ^′^^⟩ differ exactly by a single excitation *q* → *p*. Then one can write

$$\left|\Phi_{I}\rangle =C_{pq}\right|\Phi_{J}^{q\to p}\rangle$$ 25

with the cyclic permutation *C*_*pq*_ being defined in cycle notation via

$$C_{pq}=\{\begin{array}{c} (i_{p},\;i_{p}+1,\;\cdots ,\;i_{q}-1,\;i_{q})\;\mathrm{for}\;i_{p}\leq i_{q}\\ (i_{p},\;i_{p}-1,\;\cdots ,\;i_{q}+1,\;i_{q})\;\mathrm{for}\;i_{p}\geq i_{q} \end{array}$$ 26

Here, *i*_*p*_ is one of the (up to two) positions at which orbital *p* occurs in |Φ*_I_* ⟩. Using

$$AP_{\sigma }=\mathrm{sgn}\left(\sigma \right)A$$ 27

which holds for arbitrary permutations σ, one can turn eq 23 into

$$s_{pq}^{\left(k,m\right)}|\Phi_{J}^{SM}\rangle =N_{J}2^{N_{\mathrm{DOMOs}\in \left\{q\right\}}}\zeta_{pq}A\left[|\Phi_{I}\rangle C_{pq}s_{i_{q}}^{\left(k,m\right)}|X_{J}^{SM}\rangle \right]$$ 28

In this equation,

$$2^{N_{\mathrm{DOMOs}\in \{q\}}}=|S_{q}(J)|=\{\begin{array}{cc} 1 & \mathrm{if}\;q\;\mathrm{is}\;a\;\mathrm{SOMO}\\ 2 & \mathrm{if}\;q\;\mathrm{is}\;a\;\mathrm{DOMO} \end{array}$$ 29

is the number of permutations in the reduced invariance group and the sign ζ_*pq*_ is defined by^49^

$$\zeta_{pq}=\mathrm{sgn}\left(C_{pq}\right)={\left(-1\right)}^{i_{q}-i_{p}}$$ 30

Eq 30 can be understood by using eq 26 and the fact that a cyclic permutation is even if it involves an odd number of elements and vice versa. Projecting with |Φ_*I*_^*S* ^′^ *M* ^′^^⟩ from the left on eq 28 gives

$$\left\langle \Phi_{I}^{S^{\prime }M^{\prime }}\right|s_{pq}^{\left(k,m\right)}|\Phi_{J}^{SM}\rangle =N_{I}N_{J}2^{N_{\mathrm{DOMOs}\in \left\{q\right\}}}\zeta_{pq}\left[\left\langle \Phi_{I}\right|\left\langle X_{I}^{S^{\prime }M^{\prime }}\right|\right]A\left[|\Phi_{I}\rangle C_{pq}s_{i_{q}}^{\left(k,m\right)}|X_{J}^{SM}\rangle \right]$$ 31

where we used that $A^{†}A=A$ . To proceed further, one can use the double coset decomposition of *S*_*N*_ with respect to its subgroup S(I) , denoted S(I) \ $S_{N}$ / S(I) . This decomposition consists of different “double cosets” S(I)σS(I) , which are sets defined as

S(I)σS(I)={μσν|μ,ν∈S(I)} 32

for any permutation σ ∈ *S*_*N*_. The “repetition frequency” of the double coset defined in eq 32 is the number of times that the *same* element is generated by multiplying σ with *different* pairs μ, ν, i.e. it can be calculated via

$$d_{\sigma }=\frac{{\left|S\left(I\right)\right|}^{2}}{\left|S\left(I\right)\sigma S\left(I\right)\right|}$$ 33

Note that the same double coset can arise from different generating permutations σ ∈ *S*_*N*_. Any element of a double coset (which can act as a double coset “representative”) will generate the whole double coset when inserted into eq 32.

Using the decomposition of *S*_*N*_ into unique double cosets, one can write the antisymmetrizer as^49^

$$A=\frac{1}{N!}\underset{\sigma }{\sum }\frac{1}{d_{\sigma }}\underset{\mu ,\nu \in S\left(I\right)}{\sum }\mathrm{sgn}\left(\mu \sigma \nu \right)P_{\mu }P_{\sigma }P_{\nu }$$ 34

Here, the σ are the representatives chosen for the different double cosets. Note that we choose σ = 1 (identity permutation) as the representative of the double coset S(I)1S(I)=S(I) . Using $N_{I}N_{J}/N!={\left(1/\sqrt{2}\right)}^{N_{\mathrm{DOMO}}\left(I\right)+N_{\mathrm{DOMO}}\left(J\right)}$ , one obtains from eqs 31 and 34 the expression

$$\left\langle \Phi_{I}^{S^{\prime }M^{\prime }}\right|s_{pq}^{\left(k,m\right)}|\Phi_{J}^{SM}\rangle =2^{N_{\mathrm{DOMOs}\in \left\{q\right\}}}\zeta_{pq}{\left(\frac{1}{\sqrt{2}}\right)}^{N_{\mathrm{DOMO}}\left(I\right)+N_{\mathrm{DOMO}}\left(J\right)}\times \underset{\sigma }{\sum }\frac{1}{d_{\sigma }}\underset{\mu ,\nu \in S\left(I\right)}{\sum }\mathrm{sgn}\left(\mu \sigma \nu \right)\left\langle \Phi_{I}\right|P_{\mu }P_{\sigma }P_{\nu }|\Phi_{I}\rangle \times \left\langle X_{I}^{S^{\prime }M^{\prime }}\right|P_{\mu }P_{\sigma }P_{\nu }C_{pq}s_{i_{q}}^{\left(k,m\right)}|X_{J}^{SM}\rangle$$ 35

Using ⟨Φ*_I_* |*P*_μ_ *P*_σ_ *P*_ν_|Φ*_I_* ⟩ = ⟨Φ*_I_* |*P*_σ_|Φ*_I_* ⟩ = δ_σ,1_, the geminal antisymmetry of the spin functions, and *d*_1_ = $\left|S\left(I\right)\right|=2^{N_{\mathrm{DOMO}}\left(I\right)}$ , one finally obtains

$$\left\langle \Phi_{I}^{S^{\prime }M^{\prime }}\right|s_{pq}^{\left(k,m\right)}|\Phi_{J}^{SM}\rangle ={\sqrt{2}}^{N_{\mathrm{DOMOs}\in \left\{p,q\right\}}}\zeta_{pq}\left\langle X_{I}^{S^{\prime }M^{\prime }}\right|C_{pq}s_{i_{q}}^{\left(k,m\right)}|X_{J}^{SM}\rangle$$ 36

This result shows that the calculation of the matrix element of *s*_*pq*_^(*k*,*m*)^ between CSFs is reduced to the calculation of the matrix element of *C*_*pq*_ *s*_*i*_*q*__^(*k*,*m*)^ between pure spin functions. Since *C*_*pq*_ is a singlet operator, the corresponding relation of the RMEs is

$$\left\langle \Phi_{I}^{S^{\prime }}∥s_{pq}^{\left(k\right)}∥\Phi_{J}^{S}\right\rangle ={\sqrt{2}}^{N_{\mathrm{DOMOs}\in \left\{p,q\right\}}}\zeta_{pq}\left\langle X_{I}^{S^{\prime }}∥C_{pq}s_{i_{q}}^{\left(k\right)}∥X_{J}^{S}\right\rangle$$ 37

The right-hand side can be represented and evaluated using the graphical techniques of angular momentum analysis that we will introduce below (Section 2.3).

#### 2.2.2 Two-Electron Operators

The Hilbert space of two spins with *S* = 1/2 is four-dimensional. Hence, the space of operators acting on this Hilbert space has dimension 4 × 4 = 16. It is advantageous to use a basis for this operator space that consists of irreducible spin tensor operators. If we denote the rank of these operators with *k*, then the basis consists of two operators with *k* = 0, three sets of operators with *k* = 1, and one set of operators with *k* = 2. Each of these sets has 2*k* + 1 components with *m* = – *k*, – *k* + 1, ···, *k*. One can easily check that they correctly sum up to 16 components in total. An arbitrary spin tensor double excitation operator can be defined in *N*–electron space via

$$S_{pqrs}^{\left(k,m\right)}=\underset{i\neq j}{\sum }|\psi_{p}\left(r_{i}\right)\psi_{r}\left(r_{j}\right)\rangle \left\langle \psi_{q}\left(r_{i}\right)\psi_{s}\left(r_{j}\right)\right|S_{ij}^{\left(k,m\right)}$$ 38

where *S*_*ij*_^(*k*,*m*)^ is one of the irreducible two-electron pure spin operators just introduced. Any two-electron operator in a two-component relativistic picture can be expressed in terms of integrals and the 16 types of double excitation operators defined through eq 38. One of the two *k* = 0 spin operators is the identity, *S*_*ij*_^(0,0)^ = 1. In this case, eq 38 reduces to the well-known singlet double-excitation operator *e*_*pqrs*_. For *k* = 2, we write for simplicity *S*_*ij*_^(2,*m*)^ = *S*_*ij*_^*m*^ (*m* = –2, –1,0,1,2). In this case, eq 38 defines the quintet double-excitation operators that are necessary for the treatment of SSC. Note that we use a different order of orbital indices than in our previous work^47^ in order to be consistent with the order that is common practice for the *e*_*pqrs*_ operator, i.e., *S*_*pqrs*_^*m*^ = *S*_*prqs*_^*m*, prev^. Similar to the one-electron case, eq 38 is identical to a Fock space operator of the form

$$S_{pqrs}^{\left(k,m\right)}=\underset{\sigma ,\tau ,\lambda ,\nu \in \left\{\alpha ,\beta \right\}}{\sum }\left\langle \sigma \tau \right|S^{\left(k,m\right)}|\lambda \nu \rangle a_{p\sigma }^{†}a_{r\tau }^{†}a_{s\nu }a_{q\lambda }$$ 39

when considering the restriction to the FCI space. Evaluating matrix elements of eq 38 between spin-adapted CSFs proceeds along the same lines as in the one-electron case, which is why we will not present the derivation with the same level of detail as before. The action of the double excitation operator on the product of a Hartree product and a geminally antisymmetric spin function can be written as

$$S_{pqrs}^{\left(k,m\right)}\left|\Phi_{J}\rangle \right|X_{J}^{SM}\rangle =\underset{\tau \in S_{q,s}\left(J\right)}{\sum }\left(P_{\tau }C_{pqrs}^{-1}|\Phi_{I}\rangle \right)S_{\tau \left(i_{q}\right)\tau \left(i_{s}\right)}^{\left(k,m\right)}|X_{J}^{SM}\rangle =\underset{\tau \in S_{q,s}\left(J\right)}{\sum }\mathrm{sgn}\left(\tau \right)P_{\tau }\left[\left(C_{pqrs}^{-1}|\Phi_{I}\rangle \right)S_{i_{q}i_{s}}^{\left(k,m\right)}|X_{J}^{SM}\rangle \right]$$ 40

Here, $S_{q,s}\left(J\right)$ is a reduced invariance group for the Hartree product that only involves the orbitals *q* and *s* (i.e., it is the group of all permutations that only interchange DOMOs *q* and/or *s*). *i*_*q*_ is one of the (up to 2) electron positions of orbital *q* and *i*_*s*_ is one of the (up to 2) electron positions of orbital *s* in |Φ*_J_* ⟩. Eq 40 is also valid in the case *q* = *s*. In this case, a nonzero matrix element requires that *q* is a DOMO in |Φ*_J_* ⟩ and one must choose *i*_*q*_ ≠ *i*_*s*_. The permutation *C*_*pqrs*_ is defined such that

$$\left|\Phi_{I}\rangle =C_{pqrs}\right|\Phi_{J}^{qs\to pr}\rangle$$ 41

where |Φ_*J*_^*qs* → *pr*^⟩ is obtained by replacing orbital *q* in position *i*_*q*_ by orbital *p* and replacing orbital *s* in position *i*_*s*_ by orbital *r* and |Φ*_I_* ⟩ contains the same orbitals as |Φ_*J*_^*qs* → *pr*^⟩ but in canonical order. Note that, like in the one-electron case, eq 41 does not define *C*_*pqrs*_ uniquely if *p* and/or *r* are DOMOs. In this case, one can arbitrarily pick one of the up to two electron indices belonging to each DOMO and label them *i*_*p*_ and *i*_*r*_, respectively. *C*_*pqrs*_ is then uniquely defined by requiring *C*_*pqrs*_(*i*_*q*_) = *i*_*p*_ and *C*_*pqrs*_(*i*_*s*_) = *i*_*r*_.

Using eq 40, one obtains for the action of the double excitation operator on a CSF

$$S_{pqrs}^{\left(k,m\right)}\left|\Phi_{J}^{SM}\right\rangle =\left(\underset{\tau \in S_{q,s}\left(J\right)}{\sum }1\right)N_{J}\zeta_{pqrs}A\left[|\Phi_{I}\rangle C_{pqrs}S_{i_{q}i_{s}}^{\left(k,m\right)}|X_{J}^{SM}\rangle \right]=2^{N_{\mathrm{DOMOs}\in \left\{q,s\right\}}}N_{J}\zeta_{pqrs}A\left[|\Phi_{I}\rangle C_{pqrs}S_{i_{q}i_{s}}^{\left(k,m\right)}|X_{J}^{SM}\rangle \right]$$ 42

where ζ_*pqrs*_ = sgn (*C*_*pqrs*_). Like in the one-electron case, one can now use the double coset decomposition of the antisymmetrizer to obtain

$$\left\langle \Phi_{I}^{S^{\prime }M^{\prime }}\right|S_{pqrs}^{\left(k,m\right)}\left|\Phi_{J}^{SM}\right\rangle ={\sqrt{2}}^{N_{\mathrm{DOMOs}\in \left\{p,q,r,s\right\}}}\zeta_{pqrs}\left\langle X_{I}^{S^{\prime }M^{\prime }}\right|C_{pqrs}S_{i_{q}i_{s}}^{\left(k,m\right)}\left|X_{J}^{SM}\right\rangle$$ 43

The corresponding equation for the RMEs is

$$\left\langle \Phi_{I}^{S^{\prime }}∥S_{pqrs}^{\left(k\right)}∥\Phi_{J}^{S}\right\rangle ={\sqrt{2}}^{N_{\mathrm{DOMOs}\in \left\{p,q,r,s\right\}}}\zeta_{pqrs}\left\langle X_{I}^{S^{\prime }}∥C_{pqrs}S_{i_{q}i_{s}}^{\left(k\right)}∥X_{J}^{S}\right\rangle$$ 44

This is a generalization of the result derived by Wormer and Paldus for the *e*_*pqrs*_ operator^49^ to arbitrary double-excitation operators. We should note that the derivation presented in this section is not only a generalization, but also a simplification. Wormer and Paldus expressed *e*_*pqrs*_ in terms of *E*_*pq*_ operators, whereas we work directly with the definition eq 38. Furthermore, the idea of introducing reduced invariance groups $S_{q}\left(J\right)$ and $S_{q,s}\left(J\right)$ is new in our treatment and prevents one from having to explicitly enumerate the different terms that are created by the action of the excitation operator on the ket. Finally, the prefactors ${\sqrt{2}}^{N_{\mathrm{DOMOs}\in \left\{p,q\right\}}}$ and ${\sqrt{2}}^{N_{\mathrm{DOMOs}\in \left\{p,q,r,s\right\}}}$ in eqs 36 and 43 are expressed by Wormer and Paldus in a way that obscures their origin.

### 2.3 Graphical Representation of Spin Functions, Operators, and Their Matrix Elements

In this section we introduce graphical methods for manipulating angular momentum expressions and apply them to eqs 37 and 44 derived in the previous section.

#### 2.3.1 Fundamentals

The most important definitions and rules for expressing and manipulating angular momentum expressions in a graphical way are summarized in Figures 1 and 2. We exactly follow the conventions in the monograph by Brink and Satchler,^53^ to which we refer for a more in-depth discussion. The two most basic building blocks are a signed node with three lines attached to it, representing the 3*jm* symbol $\left(\begin{array}{ccc} j_{1} & j_{2} & j_{3}\\ m_{1} & m_{2} & m_{3} \end{array}\right)$ (also often called 3*j* symbol), and a line with an arrow, representing the “antisymmetric symbol”

$$\left(\begin{array}{c} j\\ m_{1},m_{2} \end{array}\right)={\left(-1\right)}^{j+m_{1}}\delta_{m_{1},-m_{2}}$$ 45

When converting an angular momentum graph into the corresponding algebraic expression, the magnetic quantum numbers of all internal lines are summed over, while the magnetic quantum numbers of external lines are variables. The order of the arguments of a 3*jm* symbol is read off from the corresponding diagram in counterclockwise fashion for a plus sign and in clockwise fashion for a minus sign. Note that each angular momentum diagram simply represents a number.

**Figure 1:**
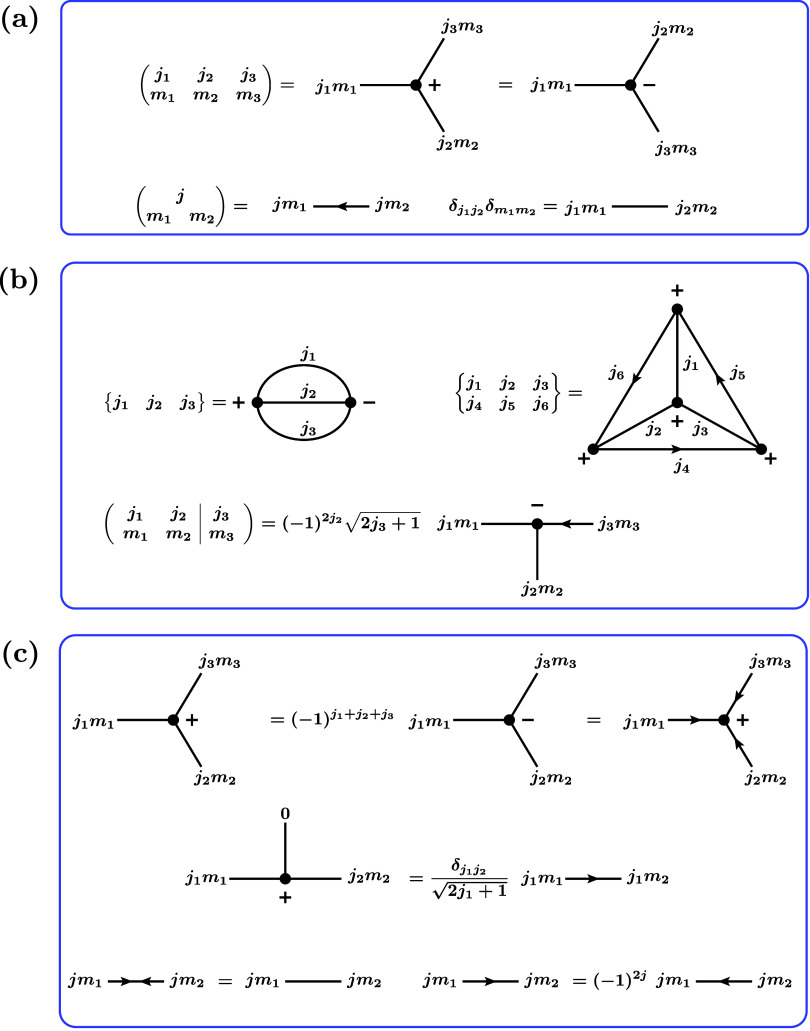
Fundamentals for expressing and manipulating angular momentum expressions graphically. (a) Equivalence of algebraic expressions and graphs for basic building blocks. (b) Equivalence of algebraic expressions and graphs for composite objects. (c) Basic rules for manipulating angular momentum graphs. Note that in the remainder of this work, an unlabeled line is tacitly assumed to be a spin 12 line.

**Figure 2:**
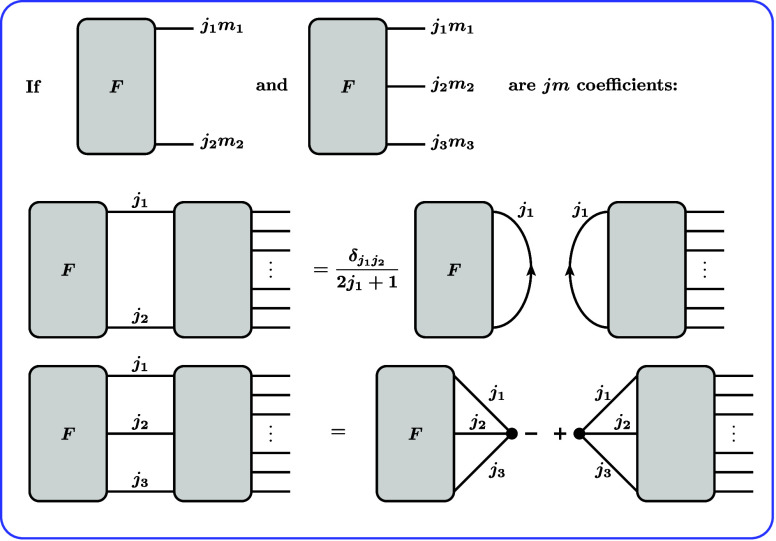
Fundamentals for expressing and manipulating angular momentum expressions graphically: Factorization rules.

The “triangular delta” (sometimes confusingly also called 3*j* symbol in analogy to the 6*j* and 9*j* symbols) displayed in Figure 1b is defined as

$$\{j_{1}j_{2}j_{3}\}=\{\begin{array}{ll} 1 & \mathrm{if}\;|j_{1}-j_{2}|\leq j_{3}\leq j_{1}+j_{2}\\ 0 & \mathrm{otherwise} \end{array}$$ 46

The graphical representation of composite objects like 6*j* symbols and CGCs are also shown in Figure 1b. Figure 1c introduces basic rules for manipulating angular momentum graphs. These rules can be used to transform graphs into some standard form such that common building blocks like 6*j* symbols can be recognized. The factorization rules in Figure 2 are only valid if the *F* block is a “*jm* coefficient”. This is the case if and only if it can be represented graphically such that every internal line has exactly one arrow and every external line has no arrow.^53^

It is also important to note that the sign factors arising when manipulating angular momentum diagrams can often be simplified by using that the three angular momenta *j*_1_, *j*_2_, *j*_3_ of a nonzero 3*jm* symbol sum up to an integer number, which means that, for example, (− 1)^2*j*_1_+2*j*_2_+2*j*_3_^ = 1.

The graphical formalism can be used to facilitate the evaluation of the matrix elements between pure spin functions like in eqs 36 and 43. An arbitrary *N*-electron pure spin function can be expanded in the 2*^N^*-dimensional tensor product basis {|*m*_1_···*m*_*N*_⟩} (with *m*_*i*_ = ± 1/2), i.e.,

$$\left|X\rangle =\underset{m_{1}\cdot \cdot \cdot m_{N}}{\sum }|m_{1}\cdot \cdot \cdot m_{N}\right\rangle \left\langle m_{1}\cdot \cdot \cdot m_{N}\right|X\rangle$$ 47

Similarly, a bra spin function can be written as

$$\langle Y|=\underset{m_{1}\cdot \cdot \cdot m_{N}}{\sum }\left\langle Y\right|m_{1}\cdot \cdot \cdot m_{N}\rangle \left\langle m_{1}\cdot \cdot \cdot m_{N}\right|$$ 48

and an operator acting on spin functions can be expressed in terms of its matrix elements,

$$O=\underset{m_{1}\cdot \cdot \cdot m_{N}}{\sum }\underset{m_{1}^{\prime }\cdot \cdot \cdot m_{N}^{\prime }}{\sum }|m_{1}\cdot \cdot \cdot m_{N}\rangle \left\langle m_{1}\cdot \cdot \cdot m_{N}\right|O|m_{1}^{\prime }\cdot \cdot \cdot m_{N}^{\prime }\rangle \left\langle m_{1}^{\prime }\cdot \cdot \cdot m_{N}^{\prime }\right|$$ 49

The coefficients ⟨*m*_1_···*m*_*N*_|*X*⟩ and ⟨*Y*|*m*_1_···*m*_*N*_⟩ and the matrix elements ⟨*m*_1_···*m*_*N*_|*O*|*m*_1_^′^···*m*_*N*_^′^⟩ completely determine the spin functions and the operator, respectively. In accordance with the papers by Drake, Kent, and Schlesinger,^43,54–56^ we represent these coefficients and operator matrix elements as shown in Figure 3: A graph with downward-facing external lines for the coefficients of a ket spin function, a graph with upward-facing external lines for the coefficients of a bra spin function, and a graph with both upward- and downward-facing external lines for the operator matrix elements. A matrix element of the form ⟨*Y*|*O*|*X*⟩ can then be expressed in terms of a contraction of coefficients,

$$\left\langle Y\right|O|X\rangle =\underset{m_{1}\cdot \cdot \cdot m_{N}}{\sum }\underset{m_{1}^{\prime }\cdot \cdot \cdot m_{N}^{\prime }}{\sum }\left\langle Y\right|m_{1}\cdot \cdot \cdot m_{N}\rangle \left\langle m_{1}\cdot \cdot \cdot m_{N}\right|O|m_{1}^{\prime }\cdot \cdot \cdot m_{N}^{\prime }\rangle \left\langle m_{1}^{\prime }\cdot \cdot \cdot m_{N}^{\prime }\right|X\rangle$$ 50

**Figure 3:**
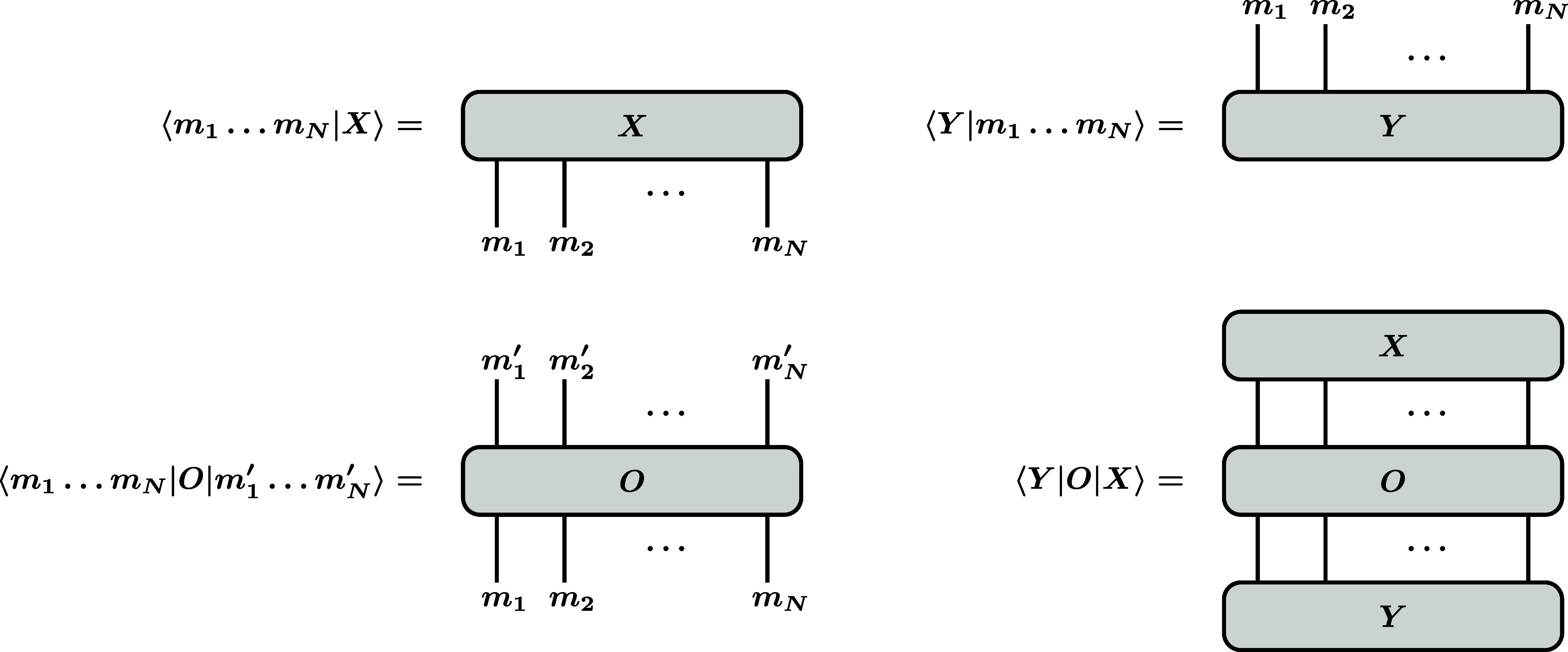
Graphical representations of spin functions (bra and ket), operators, and matrix elements.

The graphical analog of this equation is obtained by connecting the lines of the spin functions and the operator, which is also shown in Figure 3.

#### 2.3.2 Graphical Representation of Eqs 37 and 44

Equipped with the graphical techniques for angular momentum coupling, we now come back to the representation of coupling coefficient RMEs in terms of pure spin functions, eqs 37 and 44. In Appendix 1.2, we present a simple rederivation of the Wigner–Eckart theorem, which leads to an explicit expression for the reduced matrix element (eq 100). Using this equation, eqs 37 and 44 become

$$\left\langle \Phi_{I}^{S^{\prime }}∥s_{pq}^{\left(k\right)}∥\Phi_{J}^{S}\right\rangle ={\sqrt{2}}^{N_{\mathrm{DOMOs}\in \left\{p,q\right\}}}\frac{1}{2S^{\prime }+1}\zeta_{pq}\times \underset{mMM^{\prime }}{\sum }\left(\begin{array}{cc} S & k\\ M & m \end{array}|\begin{array}{c} S^{\prime }\\ M^{\prime } \end{array}\right)\left\langle X_{I}^{S^{\prime }M^{\prime }}\right|C_{pq}s_{i_{q}}^{\left(k,m\right)}|X_{J}^{SM}\rangle$$ 51

and

$$\left\langle \Phi_{I}^{S^{\prime }}∥S_{pqrs}^{\left(k\right)}∥\Phi_{J}^{S}\right\rangle ={\sqrt{2}}^{N_{\mathrm{DOMOs}\in \left\{p,q,r,s\right\}}}\frac{1}{2S^{\prime }+1}\zeta_{pqrs}\times \underset{mMM^{\prime }}{\sum }\left(\begin{array}{cc} S & k\\ M & m \end{array}|\begin{array}{c} S^{\prime }\\ M^{\prime } \end{array}\right)\left\langle X_{I}^{S^{\prime }M^{\prime }}\right|C_{pqrs}S_{i_{q}i_{s}}^{\left(k,m\right)}|X_{J}^{SM}\rangle$$ 52

Using the representation of CGCs in Figure 1b, the RMEs defined in eqs 51 and 52 can be graphically represented as shown in Figure 4. Note that we dropped the factors (−1)^2*k*^ = 1 occurring in the definition of the CGCs. They are unity since *k* can take only integer values.

**Figure 4:**
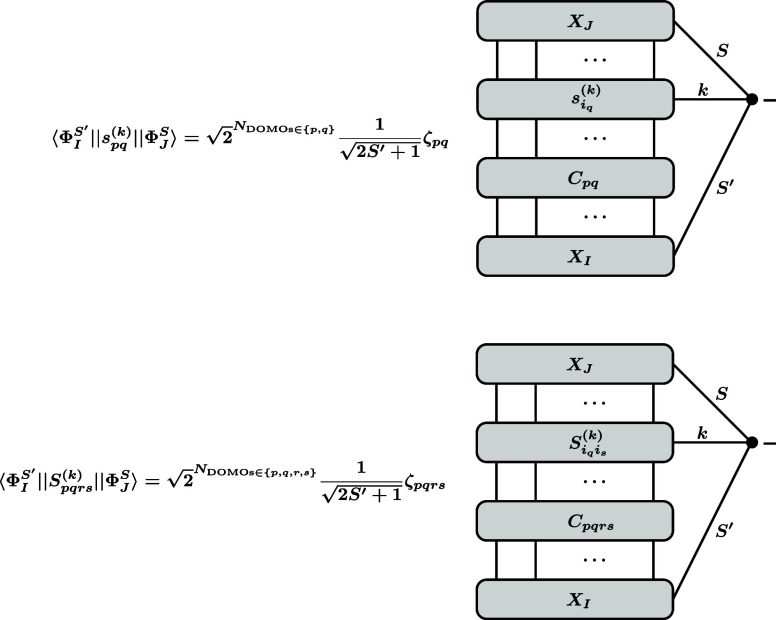
Graphical representation of the RMEs eqs 51 and 52 arising for matrix elements of single-excitation and double-excitation spin tensor excitation operators between CSFs.

#### 2.3.3 Spin Functions and Operators Relevant to This Work

So far, the treatment of Sections 2.2 and 2.3 was completely general, applying to arbitrary CSFs (with geminally antisymmetric spin functions) and arbitrary single-excitation or double-excitation spin tensor operators. From now on, we focus on YK CSFs and on the triplet single-excitation and the quintet double-excitation operators, which are needed for the treatment of SOC and SSC as detailed in Section 2.1. YK spin functions are defined by precoupling the spins of each pair of electrons associated with a DOMO to a separate singlet, and sequentially coupling the remaining electrons (associated with SOMOs) to the desired total spin *S* and magnetic quantum number *M* according to some sequence of intermediate spins *S*_*i*_. This means that in the absence of DOMOs, a YK spin function is identical to a Young–Yamanouchi (YY) spin function,^50,52^ which has the form

$$\left|X^{\mathrm{YY},SM}\rangle =\underset{m_{1}\cdot \cdot \cdot m_{N_{\mathrm{SOMO}}}}{\sum }\left[\underset{M_{1}\cdot \cdot \cdot M_{N_{\mathrm{SOMO}}-1}}{\sum }\overset{N_{\mathrm{SOMO}}}{\underset{i=1}{\prod }}\left(\begin{array}{cc} S_{i-1} & 1/2\\ M_{i-1} & m_{i} \end{array}|\begin{array}{c} S_{i}\\ M_{i} \end{array}\right)\right]\right|m_{1}\cdot \cdot \cdot m_{N_{\mathrm{SOMO}}}\rangle$$ 53

Note that *S*_*N*_SOMO__ = *S* and *M*_*N*_SOMO__ = *M*. The different linearly independent CSFs that arise from the same spatial orbital occupation correspond to the different possible chains of intermediate spins that lead to the desired total spin quantum numbers. For each pair of electrons *i* and *i* + 1 occupying the same spatial orbital (DOMO), the corresponding part of the YK spin function has the form

$$\left|X^{\mathrm{DOMO}}\rangle =\underset{m_{i},m_{i+1}}{\sum }\frac{1}{\sqrt{2}}\left(\begin{array}{c} 1/2\\ m_{i+1}m_{i} \end{array}\right)\right|m_{i}m_{i+1}\rangle =\frac{1}{\sqrt{2}}\left[\alpha \left(i\right)\beta \left(i+1\right)-\beta \left(i\right)\alpha \left(i+1\right)\right]$$ 54

The total YK spin function is the product of eq 53 and one copy of eq 54 for each DOMO. The graphical representation of these components is shown in Figure 5a, where we used the graphical form of the antisymmetric symbol and of the CGC shown respectively in Figure 1a,b.

**Figure 5:**
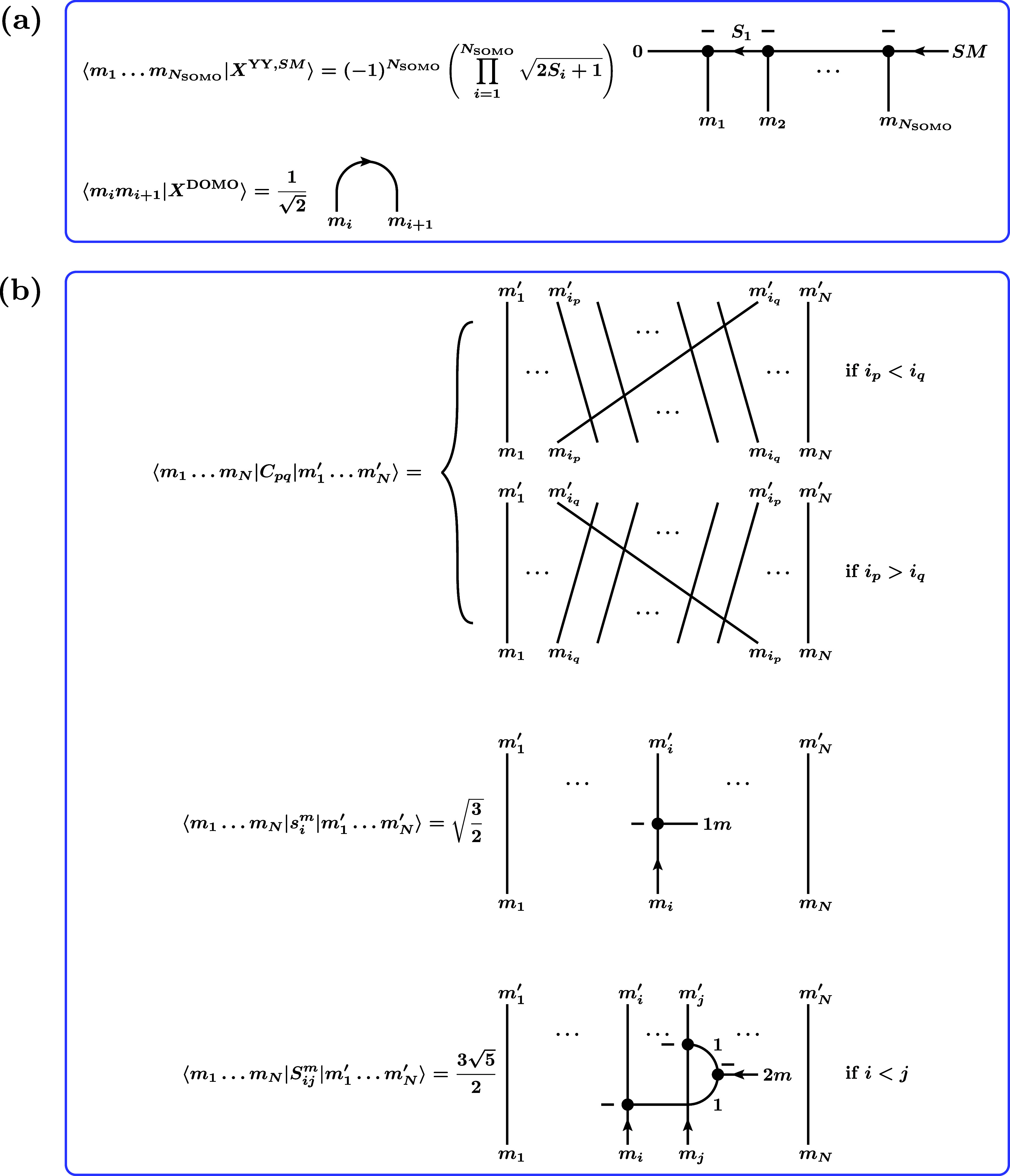
Graphical representation of spin functions and operators occurring in the present work. (a) Components of a YK spin function in graphical form: A YY spin function coupling all spins associated with SOMOs and a separate singlet spin function for each DOMO. (b) Operators needed in this work.

A permutation π ∈ *S*_*N*_ acts on a spin function via

$$P_{\pi }\left|X\rangle =\underset{m_{1}\cdots m_{N}}{\sum }P_{\pi }\right|m_{1}\cdots m_{N}\rangle \left\langle m_{1}\cdots m_{N}\right|X\rangle =\underset{m_{1}\cdots m_{N}}{\sum }\left|m_{\pi^{-1}\left(1\right)}\cdots m_{\pi^{-1}\left(N\right)}\rangle \left\langle m_{1}\cdots m_{N}\right|X\rangle =\underset{m_{1}\cdots m_{N}}{\sum }\right|m_{1}\cdots m_{N}\rangle \left\langle m_{\pi \left(1\right)}\cdots m_{\pi \left(N\right)}\right|X\rangle$$ 55

which leads to

$$\left\langle m_{1}\cdot \cdot \cdot m_{N}\right|P_{\pi }|m_{1}^{\prime }\cdot \cdot \cdot m_{N}^{\prime }\rangle =\left\langle m_{\pi \left(1\right)}\cdot \cdot \cdot m_{\pi \left(N\right)}\right|m_{1}^{\prime }\cdot \cdot \cdot m_{N}^{\prime }\rangle =\delta_{m_{\pi \left(1\right)},m_{1}^{\prime }}\cdot \cdot \cdot \delta_{m_{\pi \left(N\right)},m_{N}^{\prime }}$$ 56

This means that the graphical representation for *P*_π_ can be obtained by drawing for each *i* a line between position *i* in the ket and position π­(*i*) in the bra. The corresponding graphical representation of the cyclic permutation operator *C*_*pq*_ introduced in eq 25 is shown in Figure 5b.

The representation of the irreducible spin operator *s*_*i*_^*m*^, which is necessary when evaluating eq 51 for the RME of a triplet single-excitation operator, can be obtained as follows: Using the Wigner–Eckart theorem, a matrix element of the spin operator is easily shown to be

$$\left\langle m_{i}\right|s_{i}^{m}|m_{i}^{\prime }\rangle =-\frac{\sqrt{3}}{2}\left(\begin{array}{cc} 1 & 1/2\\ m & m_{i}^{\prime } \end{array}|\begin{array}{c} 1/2\\ m_{i} \end{array}\right)$$ 57

The representation of *s*_*i*_^*m*^ in the many-electron pure spin basis is therefore given by

$$\left\langle m_{1}\cdot \cdot \cdot m_{N}\right|s_{i}^{m}|m_{1}^{\prime }\cdot \cdot \cdot m_{N}^{\prime }\rangle =-\frac{\sqrt{3}}{2}\delta_{m_{1},m_{1}^{\prime }}\cdot \cdot \cdot \delta_{m_{i-1},m_{i-1}^{\prime }}\left(\begin{array}{cc} 1 & 1/2\\ m & m_{i}^{\prime } \end{array}|\begin{array}{c} 1/2\\ m_{i} \end{array}\right)\delta_{m_{i+1},m_{i+1}^{\prime }}\cdot \cdot \cdot \delta_{m_{N},m_{N}^{\prime }}$$ 58

The graphical equivalent of this equation follows from the graphical representation of the CGCs given in Figure 1b and is also shown in Figure 5b.

The *k* = 2 quintet spin operators *S*_*ij*_^*m*^ can be expressed by coupling the single-spin operators *s*_*i*_^*m*^,

$$S_{ij}^{m}=\underset{\gamma \gamma^{\prime }}{\sum }\left(\begin{array}{cc} 1 & 1\\ \gamma & \gamma^{\prime } \end{array}|\begin{array}{c} 2\\ m \end{array}\right)s_{i}^{\gamma }s_{j}^{\gamma^{\prime }}$$ 59

Using this and eq 57, the matrix representation is given by

$$\left\langle m_{1}\cdot \cdot \cdot m_{N}\right|S_{ij}^{m}|m_{1}^{\prime }\cdot \cdot \cdot m_{N}^{\prime }\rangle =\frac{3}{4}\underset{\gamma \gamma^{\prime }}{\sum }\left(\begin{array}{cc} 1 & 1\\ \gamma & \gamma^{\prime } \end{array}|\begin{array}{c} 2\\ m \end{array}\right)\left[\delta_{m_{1},m_{1}^{\prime }}\cdot \cdot \cdot \left(\begin{array}{cc} 1 & 1/2\\ \gamma & m_{i}^{\prime } \end{array}|\begin{array}{c} 1/2\\ m_{i} \end{array}\right)\cdot \cdot \cdot \left(\begin{array}{cc} 1 & 1/2\\ \gamma & m_{j}^{\prime } \end{array}|\begin{array}{c} 1/2\\ m_{j} \end{array}\right)\cdot \cdot \cdot \delta_{m_{N},m_{N}^{\prime }}\right]$$ 60

whose graphical representation is also shown in Figure 5b.

There exist alternative graphical representations of the *S*_*ij*_^*m*^ operator that will turn out to be useful for our purposes. They can be understood via a connection to the state of four spins (each with *S* = 1/2) coupled to a total quintet. The *S* = 2 state

$$\left|2M\rangle =|\left(\left(\frac{1}{2}\frac{1}{2}\right)1,\;\left(\frac{1}{2}\frac{1}{2}\right)1\right)2M\right\rangle$$ 61

that is obtained by first coupling each of two pairs of spins to a triplet and then coupling the two intermediate triplets to the total quintet, can be written in graphical form as shown in Figure 6a.When comparing this graph with the one of the *S* ^*m*^ operator in Figure 5b, it becomes clear that one can obtain the matrix elements of the quintet spin operator by taking the graphical representation of the coefficient of the *S* = 2 state (eq 61), dividing it by 2, and attaching an inward-facing arrow to the *S* = 2 line and two of the external *S* = 1/2 lines. This procedure can be written algebraically as

$$\left\langle m_{i}m_{j}\right|S_{ij}^{m}|m_{i}^{\prime }m_{j}^{\prime }\rangle =\frac{1}{2}\underset{m_{1}m_{3}M}{\sum }\left(\begin{array}{c} 1/2\\ m_{1},m_{i} \end{array}\right)\left(\begin{array}{c} 1/2\\ m_{3},m_{j} \end{array}\right)\left(\begin{array}{c} 2\\ M,m \end{array}\right)\left\langle m_{1}m_{i}^{\prime }m_{3}m_{j}^{\prime }\right|2M\rangle$$ 62

**Figure 6:**
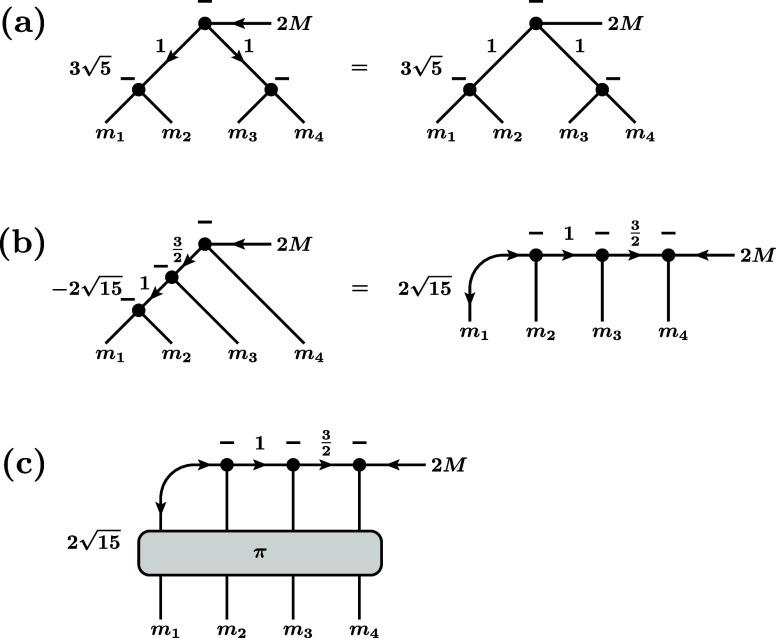
Three alternative graphical representations of the coefficient ⟨*m*_1_ *m*_2_ *m*_3_ *m*_4_ | 2*M*⟩ of the quintet state |2*M*⟩ that results from coupling four spins with *S* = 1/2. (a) Graphical representation corresponding to a precoupling to two triplets, $\left|\left(\left(\frac{1}{2}\frac{1}{2}\right)1,\;\left(\frac{1}{2}\frac{1}{2}\right)1\right)2M\right\rangle$ . (b) Graphical representation corresponding to a sequential coupling, $\left|\left(\left(\left(\frac{1}{2}\frac{1}{2}\right)1,\;\frac{1}{2}\right)\frac{3}{2},\;\frac{1}{2}\right)2M\right\rangle$ . (c) Graphical representation corresponding to a sequential coupling followed by an arbitrary permutation, $P_{\pi }\left|\left(\left(\left(\frac{1}{2}\frac{1}{2}\right)1,\;\frac{1}{2}\right)\frac{3}{2},\;\frac{1}{2}\right)2M\right\rangle$ .

Our central insight is now that there is only a *single S* = 2 state that can be formed from four spins with *S* = 1/2, meaning that *any* coupling scheme must lead to exactly the *same S* = 2 state. One can then use the graphical representation of the *S* = 2 state coefficient according to an arbitrary coupling scheme, apply the procedure of eq 62, and obtain a valid graphical representation of the *S* ^*m*^ operator. Incidentally, this approach straightforwardly leads to the statement *S* ^*m*^ *P* = *PS* ^*m*^ = *S* ^*m*^ (where *P* is the permutation operator of the two spins) that we derived previously on a case-by-case basis^47^ in order to prove the “permutational relation” ^47,48^ (eq 13). Of particular importance for our purposes is the sequential coupling of the four spins to *S* = 1, then *S* = 3/2, and finally *S* = 2, |2*M*⟩ = $|\left(\left(\left(\frac{1}{2}\frac{1}{2}\right)1,\;\frac{1}{2}\right)\frac{3}{2},\;\frac{1}{2}\right)2M\rangle$ , which is represented by the graph in Figure 6b.

It is also important to note that the state |2*M*⟩ is *symmetric* under arbitrary permutations, i.e., *P*_π_ | 2*M*⟩ = | 2*M*⟩ for any of the 4 ! = 24 permutations π ∈ *S*_4_ of the symmetric group. This leads to the representation in Figure 6c. This representation can also be interpreted in terms of the fact that the four spins can be sequentially coupled in *any* arbitrary order to yield the same quintet state.

It is advantageous to consider the *S*_*ij*_^*m*^ operator not in isolation but in the context of the combined operator *C*_*pqrs*_ *S*_*i*_*q*_ *i*_*s*__^*m*^ occurring in Figure 4. The permutation *C*_*pqrs*_ essentially just moves *i*_*q*_ into *i*_*p*_ and *i*_*s*_ into *i*_*r*_ (shifting all other positions accordingly), i.e., according to eq 56 its matrix representation contains the part $\delta_{m_{C_{pqrs}\left(i_{q}\right),m_{i_{q}}^{\prime }}}\delta_{m_{C_{pqrs}\left(i_{s}\right),m_{i_{s}}^{\prime }}}=\delta_{m_{i_{p}},m_{i_{q}}^{\prime }}\delta_{m_{i_{r}},m_{i_{s}}^{\prime }}$ . A contraction of this expression with the matrix representation of the *S*_*i*_*q*_ *i*_*s*__^*m*^ operator essentially just changes the label *m*_*i*_q__ to *m*_*i*_p__ and the label *m*_*i*_s__ to *m*_*i*_r__. Combining this insight with eq 62 and the representation of the quintet state given in Figure 6c, we obtain the graphical representation of the combined operator *C*_*pqrs*_ *S*_*i*_*q*_ *i*_*s*__^*m*^ shown in Figure 7.

**Figure 7:**
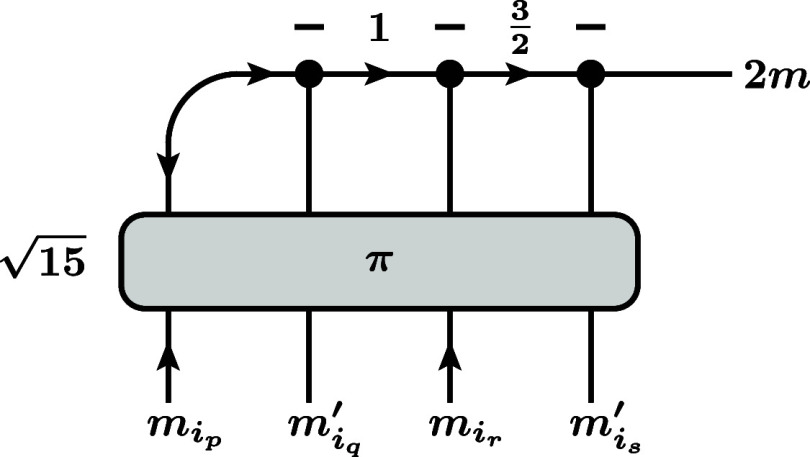
Graphical representation of the combined operator ⟨*m*_1_···*m*_*N*_ | *C*_*pqrs*_ *S*_*i*_*q*_ *i*_*s*__^*m*^ | *m*_1_^′^···*m*_*N*_^′^⟩. Note that π can be an arbitrary permutation of the four lines, i.e., the lines at the bottom of the π block can be arbitrarily connected to the lines at the top of the block. Also note that for simplicity we did not draw the factor $\underset{i\neq i_{q},i_{s}}{\prod }\delta_{m_{C_{pqrs}\left(i\right)},m_{i}^{\prime }}$ , where the Kronecker deltas are simply represented by straight lines.

Given a specific set of electron indices *i*_*p*_, *i*_*q*_, *i*_*r*_, *i*_*s*_, one can now choose the permutation π in Figure 7 such that the corresponding lines will be sequentially coupled in order of increasing indices. This will be advantageous in the following for the decomposition of the RMEs into factors. Under the constraints *i*_*p*_ < *i*_*r*_ and *i*_*q*_ < *i*_*s*_ that were discussed in Section 2.1.2, there remain six relevant permutations out of the total 4!=24. After turning the ket lines to the top, which changes the minus sign on the respective node into a plus sign, one obtains the equivalent graphical representations shown in Figure 8.

**Figure 8:**
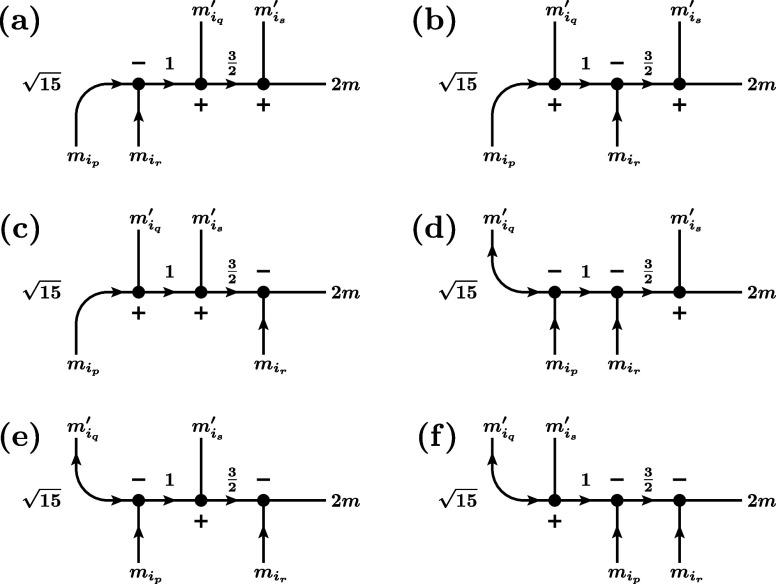
Alternative representations of the combined operator ⟨*m*_1_···*m*_*N*_ | *C*_*pqrs*_ *S*_*i*_*q*_ *i*_*s*__^*m*^ | *m*_1_^′^···*m*_*N*_^′^⟩. Note that the six shown representations (out of 4!=24 possible ones) correspond to the restriction that *m*_*i*_p__ should occur before *m*_*i*_r__ and *m*_*i*_*q*__^′^ before *m*_*i*_*s*__^′^ in the sequential coupling. Also note that, like in Figure 7, we did not draw the product of Kronecker deltas $\underset{i\neq i_{q},i_{s}}{\prod }\delta_{m_{C_{pqrs}\left(i\right)},m_{i}^{\prime }}$ that are also a part of the matrix element. (a) Order *prqs*. (b) Order *pqrs*. (c) Order *pqsr*. (d) Order *qprs*. (e) Order *qpsr*. (f) Order *qspr*.

### 2.4 Working Equations for Triplet Single-Excitation and Quintet Double-Excitation Operators and YK CSFs

It can easily be shown that an orbital that is a DOMO in both bra and ket CSF and not involved in the excitation, will not contribute to the RME of an excitation operator. We therefore remove all such redundant DOMOs before calculating the RME. Since orbitals that are unoccupied in both bra and ket also do not contribute to the total matrix element, these virtual MOs (VMOs) are dropped as well. In the following, we will use the symbol *n* to denote the total number of orbitals after removing those that are doubly occupied or unoccupied in both bra and ket and not involved in the excitation. We furthermore note that the number of SOMOs in the bra and ket CSF is either both even (for an integer spin system) or both odd (for a half-integer spin system). Therefore, the factors (− 1)^*N*_SOMO_^ appearing in the graphical representation of the YK spin functions in Figure 5a cancel when calculating matrix elements. Finally, the prefactors ${\sqrt{2}}^{N_{\mathrm{DOMOs}\in \left\{p,q\right\}}}$ and ${\sqrt{2}}^{N_{\mathrm{DOMOs}\in \left\{p,q,r,s\right\}}}$ in Figure 4 exactly cancel the $1/\sqrt{2}$ prefactor according to eq 54 and Figure 5a for the remaining DOMOs involved in the excitation.

In the following, we will not label intermediate spins with an electron index, as done in Section 2.3.3 and some of the early literature,^43,54–56^ but with an orbital index, as is also done in the later work of Kent and Schlesinger on spin-dependent operators.^57,58^ This means that we define *S*_*t*_ = *S*_*i*_ if *t* is the *i*th SOMO (*S*_*i*_ being the intermediate spins in eq 53) and *S*_*t*_ = *S*_*t*–1_ if *t* is a DOMO or VMO. We do this because the equations resulting from this choice are more suited for an orbital-based implementation.

#### 2.4.1 Graphical Representation of the RMEs

##### 2.4.1.1 RMEs for Triplet Single-Excitation Operators

For the triplet single-excitation operator, one can distinguish three different cases, whose RMEs can be obtained in graphical form from Figure 4 and the representation of the states and operators in Figure 5. The resulting graphs are displayed in Figure 9. Note that in these graphs we align equal orbital indices (not electron indices) in the bra and the ket above each other. This means that lines that are slanted in the *C*_*pq*_ diagrams of Figure 5b become vertical lines in the RME diagrams. For the case *p* = *q*, the matrix element can only be nonzero if *p* is a SOMO. The four different blocks occurring in Figure 9 are defined in Figure 10. Note that an *A* or *Ã* block corresponds to an orbital that is *annihilated* when going from the ket to the bra, while a *B* or *B̃* block corresponds to an orbital that is *created* when going from the ket to the bra. To guide the eye, we use different colors for these types of blocks. In Section 2.2, we mentioned that the definition of the electron indices *i*_*p*_, *i*_*q*_, *i*_*r*_, and *i*_*s*_ is ambiguous in the case of DOMOs and that any choice is possible as long as it is used consistently. In Figure 10, we fix this choice by defining *i*_*p*_ and *i*_*r*_ (occurring in *B* or *B̃* blocks) to be the *first* occurrence of an orbital (*left* line in the diagram), and *i*_*q*_ and *i*_*s*_ (occurring in *A* or *Ã* blocks) to be the *second* occurrence of an orbital (*right* line in the diagram) in the case of DOMOs.

**Figure 9:**
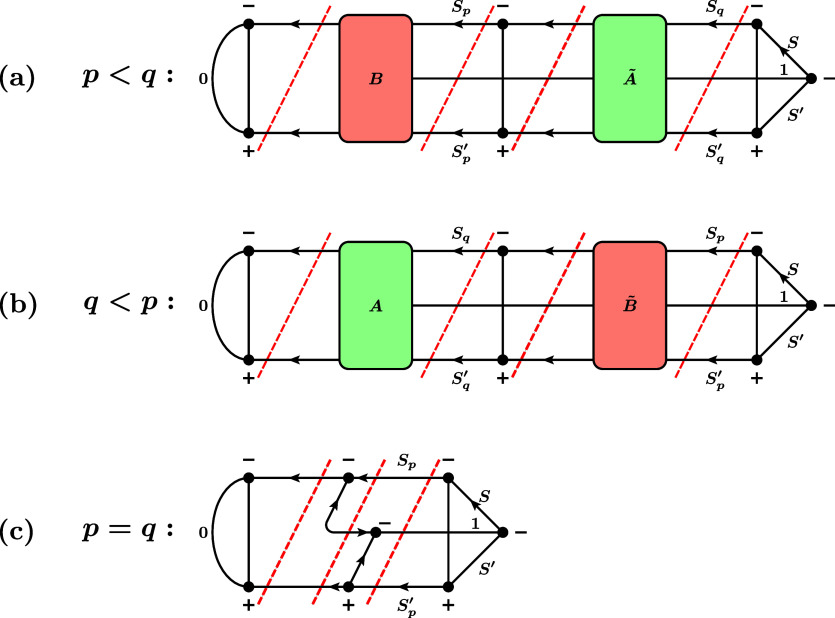
Three possible cases for the RMEs of the triplet single-excitation operator. (a) *p* < *q*, (b) *q* < *p*, (c) *p* = *q*. For simplicity, the prefactor multiplying these graphs to obtain the actual RME, $\sqrt{\frac{3}{2}}\frac{1}{\sqrt{2S^{\prime }+1}}\zeta_{pq}\underset{t\in \mathrm{SOMOs}\left(I,J\right)}{\prod }\sqrt{\left(2S_{t}+1\right)\left(2S_{t}^{\prime }+1\right)}$ , is not drawn. Here, SOMOs­(*I*, *J*) denotes the set of orbitals that are SOMOs in both the bra *I* and the ket *J*. Furthermore, we draw the case of exactly one SOMO pair before and after orbitals *p* and *q*. In general, any number (also zero) is possible. The red dashed lines denote the positions where the RMEs are cut into factors according to the rules in Figure 2.

**Figure 10:**
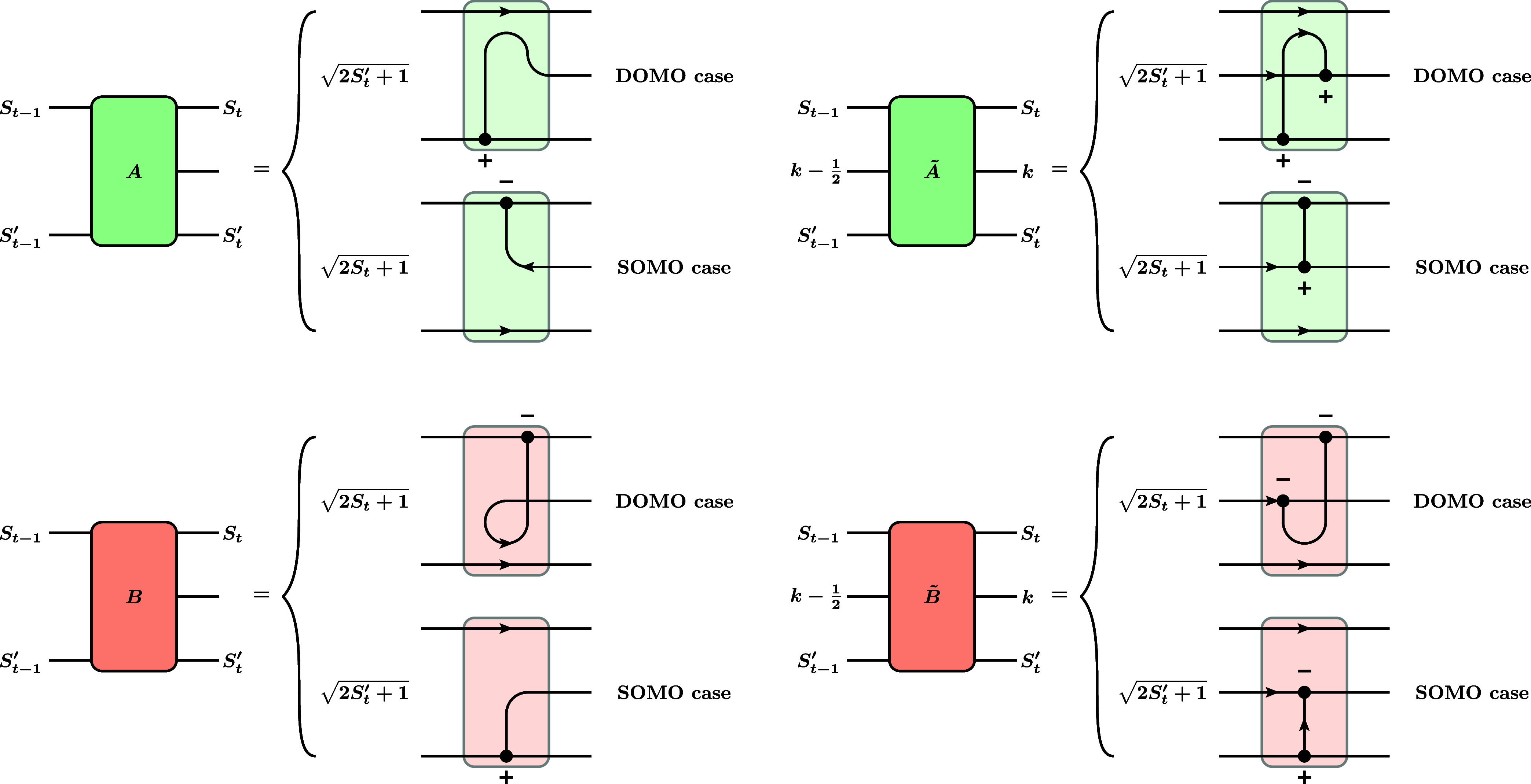
Definition of the *A*, *B*, *Ã*, and *B̃* blocks occurring in the different cases of RMEs for the triplet single-excitation and quintet double-excitation operator. “DOMO case” means that the orbital *t* is a DOMO in the ket for *A* or *Ã* blocks, and a DOMO in the bra for *B* or *B̃* blocks, respectively. “SOMO case” is defined analogously.

The spin diagram for the *p* > *q* case of the triplet single-excitation operator was also given by Drake and Schlesinger in their original work.^43^ However, our treatment differs in a number of points: We use a different convention for the Wigner–Eckart theorem and the corresponding RME than the Racah–Wigner convention used by Drake and Schlesinger, which leads to a different prefactor. Furthermore, Drake and Schlesinger employed inverted counting (*S*_0_ being the total spin after all SOMOs are coupled) and labeled intermediate spins by electron indices instead of orbital indices. Because of this choice, they need to define a larger number of different factors than strictly necessary.

##### 2.4.1.2 RMEs for Quintet Double-Excitation Operators

We now consider the case of the *k* = 2 (quintet) double-excitation operator *S*_*pqrs*_^*m*^. This operator has a few properties that simplify its treatment. From the permutational relation eq 13, it follows immediately that the *S*_*pqrs*_^*m*^ operators are zero unless they satisfy the “selection rules” *p* ≠ *r* and *q* ≠ *s*.^47,48^ Furthermore, “closed shells do not contribute” ^47,48^ to the matrix elements (i.e., if *p* = *q* and/or *r* = *s*, the corresponding orbital must be a SOMO for the matrix element to be nonzero). Using this fact as well as the selection rules, one can distinguish a total of 13 different cases with *p* < *r* and *q* < *s*, which are summarized in Table 1. The graphical representations of the corresponding RMEs, using Figures 4, 5a, and 8, are shown in Figures 11 and 12. Out of the different possibilities displayed in Figure 8, we always use the graphical representation of the *C*_*pqrs*_ *S*_*i*_*q*_ *i*_*s*__^*m*^ operator that leads to the appearance of a sequentially coupled sequence of spins from the left to the right of the diagram. We also indicate this choice in the last column of Table 1. Also note that in the case of equalities among the orbitals *p*, *q*, *r*, and *s*, we make the (arbitrary) choice that the operator first couples to the ket and then to the bra.

**Table 1:**
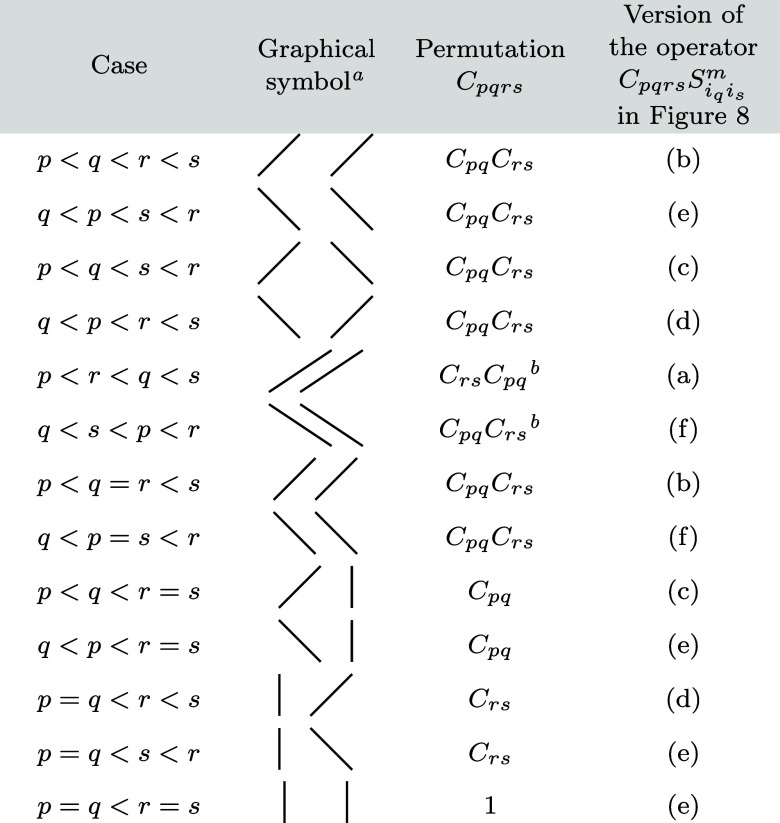
Overview of the Different Cases Occurring for the Quintet Double Excitation Operator

a The graphical symbols visualize the positions of the *pq* orbital pair (left line, *p* at the bottom and *q* at the top) and the *rs* orbital pair (right line, *r* at the bottom and *s* at the top) within the set of all orbitals. Note that the lines cannot cross because of the conditions *p* < *r* and *q* < *s.*

b In those cases where the ranges of *C*_*pq*_ and *C*_*rs*_ overlap, these two cyclic permutations do not commute, i.e., the order written down here is significant.

**Figure 11:**
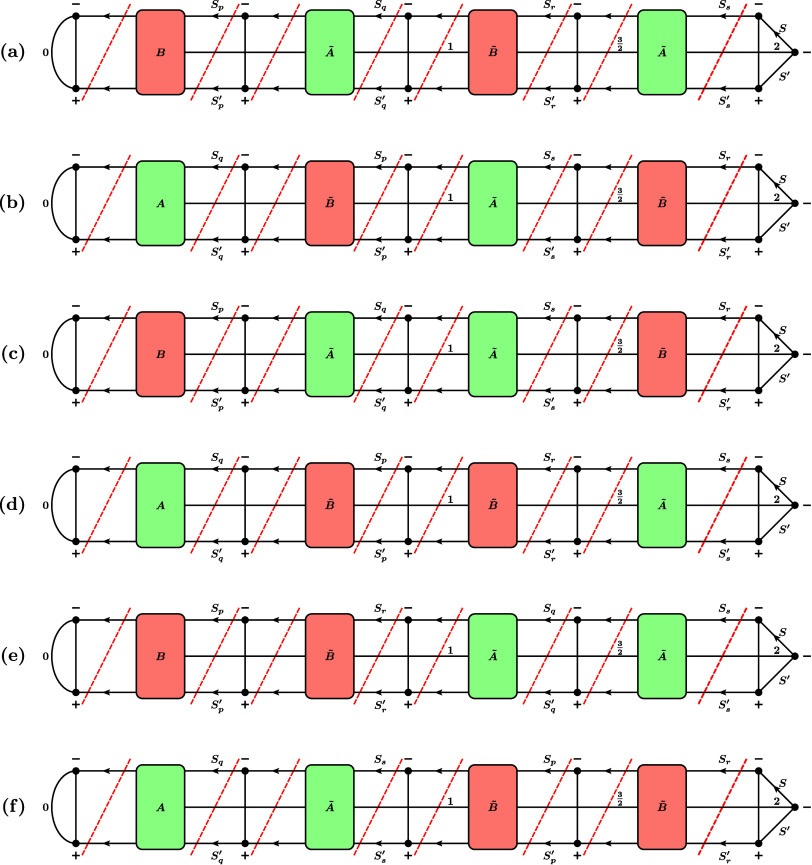
First six (out of 13) possible cases for the RMEs of the quintet double-excitation operator, involving only unique orbitals *p*, *q*, *r*, *s*. (a) *p* < *q* < *r* < *s*, (b) *q* < *p* < *s* < *r*, (c) *p* < *q* < *s* < *r*, (d) *q* < *p* < *r* < *s*, (e) *p* < *r* < *q* < *s*, (f) *q* < *s* < *p* < *r*. For simplicity, the prefactor multiplying these graphs to obtain the actual RME, $\sqrt{15}\frac{1}{\sqrt{2S^{\prime }+1}}\zeta_{pqrs}\underset{t\in \mathrm{SOMOs}\left(I,J\right)}{\prod }\sqrt{\left(2S_{t}+1\right)\left(2S_{t}^{\prime }+1\right)}$ , is not drawn. Here, SOMOs­(*I*, *J*) denotes the set of orbitals that are SOMOs in both the bra *I* and the ket *J*. Note that we draw the case of exactly one SOMO pair before and after orbitals *p*, *q*, *r*, *s*. In general, any number (also zero) is possible. The red dashed lines denote the positions where the RMEs are cut into factors according to the rules in Figure 2.

**Figure 12:**
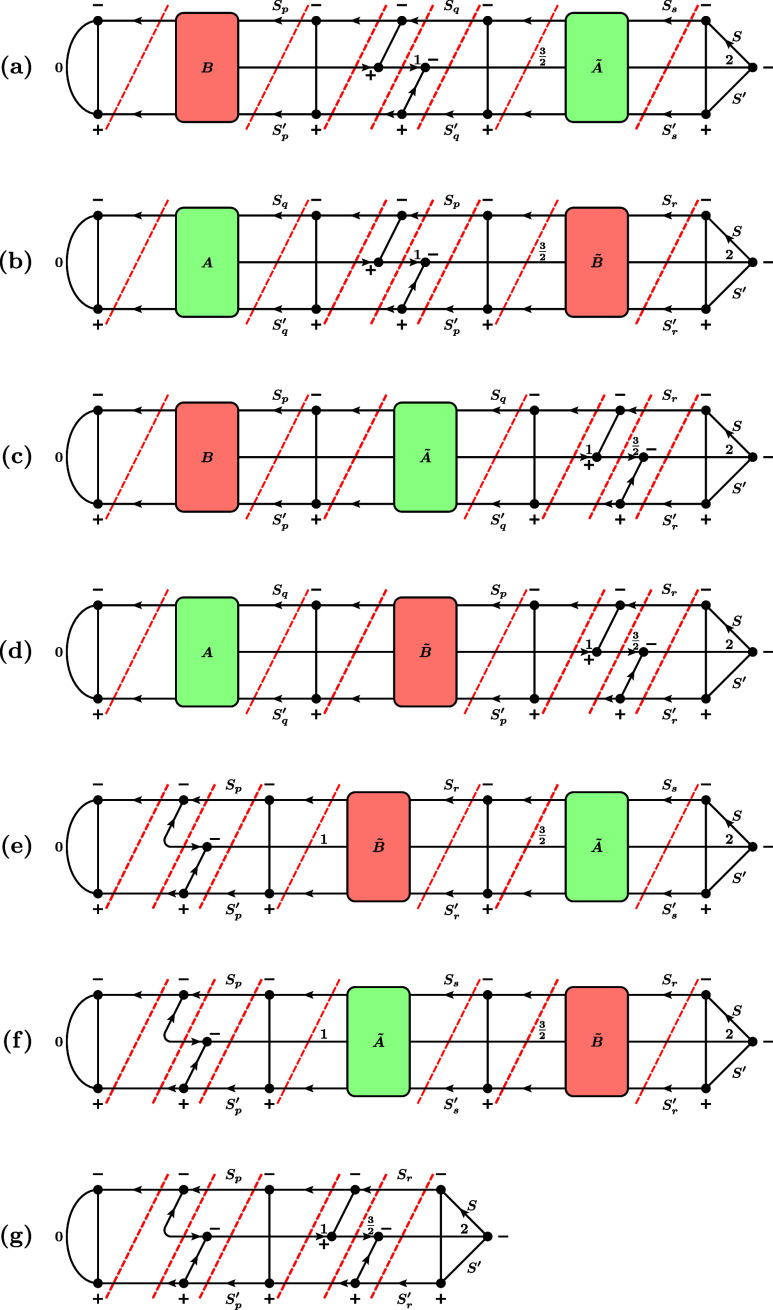
Last seven (out of 13) possible cases for the RMEs of the quintet double-excitation operator, involving equalities among the orbitals *p*, *q*, *r*, *s*. (a) *p* < *q* = *r* < *s*, (b) *q* < *p* = *s* < *r*, (c) *p* < *q* < *r* = *s*, (d) *q* < *p* < *r* = *s*, (e) *p* = *q* < *r* < *s*, (f) *p* = *q* < *s* < *r*, (g) *p* = *q* < *r* = *s*. For simplicity, the prefactor multiplying these graphs to obtain the actual RME, $\sqrt{15}\frac{1}{\sqrt{2S^{\prime }+1}}\zeta_{pqrs}\underset{t\in \mathrm{SOMOs}\left(I,J\right)}{\prod }\sqrt{\left(2S_{t}+1\right)\left(2S_{t}^{\prime }+1\right)}$ , is not drawn. Here, SOMOs­(*I*, *J*) denotes the set of orbitals that are SOMOs in both the bra *I* and the ket *J*. Note that we draw the case of exactly one SOMO pair before and after orbitals *p*, *q*, *r*, *s*. In general, any number (also zero) is possible. The red dashed lines denote the positions where the RMEs are cut into factors according to the rules in Figure 2.

Our reasoning so far was that, because of the permutational relation, we only need to consider the 13 cases arising under the constraint *p* < *r*, *q* < *s*. However, it would be reassuring if the permutational relation could also be *derived* from the present approach. This is indeed the case. We assume like before that *p* < *r* and *q* < *s*, but now we consider the matrix element of the double-excitation operator *S*_*psrq*_^*m*^ (i.e., *q* and *s* are interchanged). In this case, we should employ a graphical representation of the *S* ^*m*^ operator that differs from the one used for the *S*_*pqrs*_^*m*^ operator by switching the order in which *m*_*i*_*q*__^′^ and *m*_*i*_*s*__^′^ appear in the sequential coupling. Then one can see that the graphical representations of the RMEs for the two operators are actually *identical*. The only difference is in the sign factor: The permutations are related via *C*_*psrq*_ = *C*_*pqrs*_(*i*_*q*_ *i*_*s*_), i.e., one first has to swap the orbitals *q* and *s* before one can apply the original permutation operator; see also Wormer and Paldus’s discussion of Coulomb versus exchange matrix elements for the *e*_*pqrs*_ operator.^49^ Since a simple transposition (*i*_*q*_ *i*_*s*_) has a negative sign, it follows that ζ_*psrq*_ = – ζ_*pqrs*_, which leads to a change of sign for the *S*_*psrq*_^*m*^ matrix element compared to the *S*_*pqrs*_^*m*^ matrix element. This is exactly one of the permutational relations (eq 13). For interchanging *p* and *r* instead of *q* and *s*, the argument is analogous.

#### 2.4.2 Factorization of RMEs

After factorizing the RMEs according to the factorization rules in Figure 2 and along the positions indicated by the red dashed lines in Figures 9, 11, and 12, they can be written as a product of Kronecker deltas and the factors defined in Figure 13.

**Figure 13:**
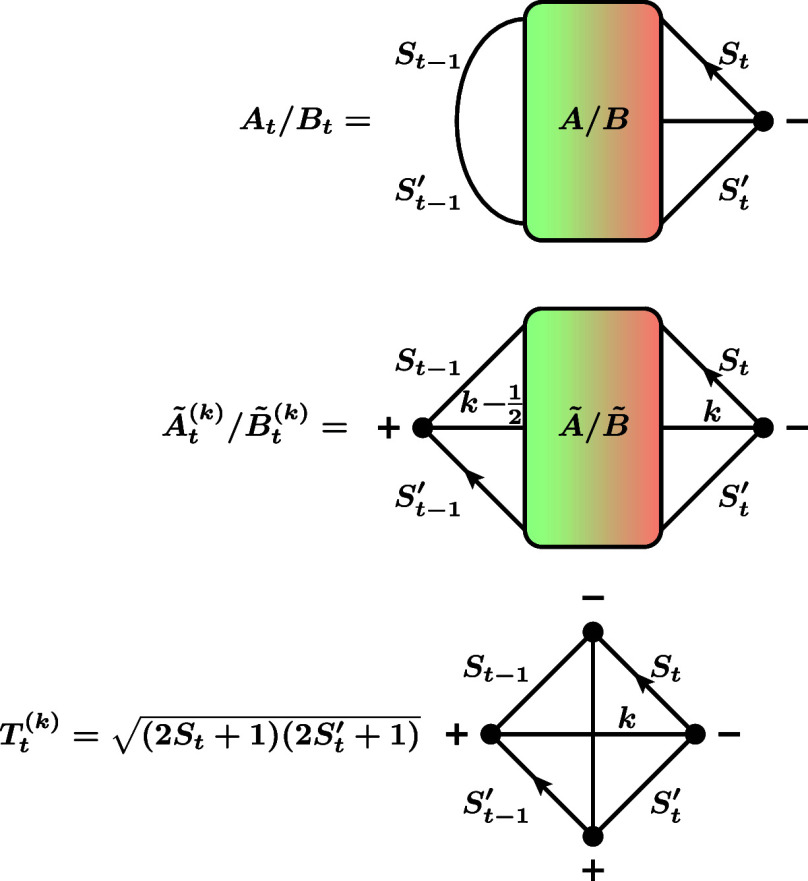
Definition of the factors needed to evaluate RMEs of triplet single-excitation and quintet double-excitation operators. See Figure 10 for the definition of the colored blocks.

As one can see from Figures 10 and 13, there is a symmetry between the factors: The SOMO case of an *A* or *Ã* factor is equal to the DOMO case of a *B* or *B̃* factor and vice versa. Using the graph manipulation rules from Figure 1c, one can express the factors in terms of a triangular delta (for *A*_*t*_ and *B*_*t*_) or a 6*j* symbol (for *Ã*_*t*_^(*k*)^, *B̃*_*t*_^(*k*)^, and *T*_*t*_^(*k*)^), whose graphical representations are shown in Figure 1b. This leads to the following algebraic equations for the factors:

$$A_{t}^{\mathrm{DOMO}}=B_{t}^{\mathrm{SOMO}}=\sqrt{2S_{t}^{\prime }+1}$$ 63

$$A_{t}^{\mathrm{SOMO}}=B_{t}^{\mathrm{DOMO}}={\left(-1\right)}^{S_{t-1}^{\prime }-S_{t}-1/2}\sqrt{2S_{t}+1}$$ 64

$${Ã}_{t}^{\left(k\right),\mathrm{DOMO}}={\mathrm{B̃}}_{t}^{\left(k\right),\mathrm{SOMO}}={\left(-1\right)}^{S_{t}^{\prime }+S_{t}-k-1}\sqrt{2S_{t}^{\prime }+1}\left\{\begin{array}{ccc} k & \frac{1}{2} & k-\frac{1}{2}\\ S_{t-1}^{\prime } & S_{t} & S_{t}^{\prime } \end{array}\right\}$$ 65

$${Ã}_{t}^{\left(k\right),\mathrm{SOMO}}={\mathrm{B̃}}_{t}^{\left(k\right),\mathrm{DOMO}}={\left(-1\right)}^{S_{t-1}^{\prime }+S_{t-1}-k+1/2}\sqrt{2S_{t}+1}\left\{\begin{array}{ccc} k & \frac{1}{2} & k-\frac{1}{2}\\ S_{t-1} & S_{t-1}^{\prime } & S_{t} \end{array}\right\}$$ 66

$$T_{t}^{\left(k\right)}={\left(-1\right)}^{S_{t-1}^{\prime }+S_{t}+k+1/2}\sqrt{\left(2S_{t}+1\right)\left(2S_{t}^{\prime }+1\right)}\left\{\begin{array}{ccc} k & S_{t}^{\prime } & S_{t}\\ \frac{1}{2} & S_{t-1} & S_{t-1}^{\prime } \end{array}\right\}$$ 67

Note that for *A*_*t*_^SOMO^ and *Ã*_*t*_^(*k*), SOMO^, one has *S*_*t*_^′^ = *S*_*t*–1_^′^, and for *B*_*t*_^SOMO^ and *B̃*_*t*_^(*k*), SOMO^, one has *S*_*t*_ = *S*_*t*–1_. However, it is very important that we only employ *S*_*t*–1_^′^ in eqs 64 and 66 and only employ *S*_*t*_ in eq 65. The reason is our chosen order in which the operators couple to the ket and the bra in the case of equalities among the orbitals *p*, *q*, *r*, and *s* (first ket, then bra); see below. The five types of factors in eqs 63–67 are everything that is needed for expressing any RME of triplet single-excitation or quintet double-excitation operators. Actually, the *A*_*t*_ and *B*_*t*_ factors are not even strictly necessary. As shown in Figure 14, the *A* and *B* blocks can be completely eliminated from the spin graphs of the RMEs, meaning that everything can be expressed in terms of only three types of factors. However, although formally simpler, this is not convenient in practice since in this representation of the RMEs, the square-root factors originating from the CGCs in the YK spin functions are assigned to factors in a different way, which leads to nontrivial factors even before the first orbital difference. This can be understood by noticing that the factor *T*_*t*_^(0)^ that would appear in the formally simplified representation of the RME is *not* just a Kronecker delta. Therefore, we keep the original version of the RME graphs, which means that the equations involve the *A*_*t*_ and *B*_*t*_ factors.

**Figure 14:**
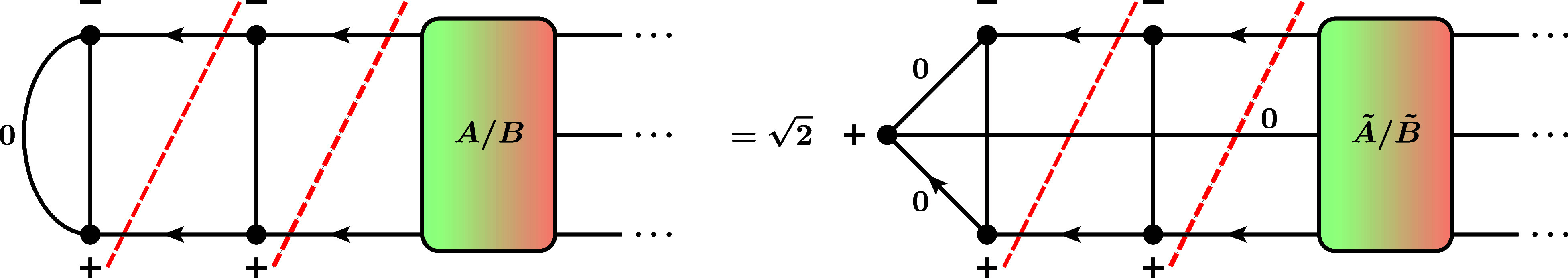
Possible elimination of *A* and *B* blocks from the RMEs.

After factorization, the equations for the RMEs of the triplet single-excitation operators (Figure 9) can be written down in terms of the factors just defined,

$$p<q:\left\langle \Phi_{I}^{S^{\prime }}∥s_{pq}∥\Phi_{J}^{S}\right\rangle =\sqrt{\frac{3}{2}}\frac{1}{\sqrt{2S^{\prime }+1}}\zeta_{pq}\left(\overset{p-1}{\underset{t=1}{\prod }}\delta_{S_{t},S_{t}^{\prime }}\right)B_{p}\left(\overset{q-1}{\underset{t=p+1}{\prod }}T_{t}^{\left(1/2\right)}\right){Ã}_{q}^{\left(1\right)}\left(\overset{n}{\underset{t=q+1}{\prod }}T_{t}^{\left(1\right)}\right)$$ 68

$$q<p:\left\langle \Phi_{I}^{S^{\prime }}∥s_{pq}∥\Phi_{J}^{S}\right\rangle =\sqrt{\frac{3}{2}}\frac{1}{\sqrt{2S^{\prime }+1}}\zeta_{pq}\left(\overset{q-1}{\underset{t=1}{\prod }}\delta_{S_{t},S_{t}^{\prime }}\right)A_{q}\left(\overset{p-1}{\underset{t=q+1}{\prod }}T_{t}^{\left(1/2\right)}\right){\mathrm{B̃}}_{p}^{\left(1\right)}\left(\overset{n}{\underset{t=p+1}{\prod }}T_{t}^{\left(1\right)}\right)$$ 69

$$p=q:\left\langle \Phi_{I}^{S^{\prime }}∥s_{pq}∥\Phi_{J}^{S}\right\rangle =\sqrt{\frac{3}{2}}\frac{1}{\sqrt{2S^{\prime }+1}}\left(\overset{p-1}{\underset{t=1}{\prod }}\delta_{S_{t},S_{t}^{\prime }}\right)A_{p}^{\mathrm{SOMO}}{\mathrm{B̃}}_{p}^{\left(1\right),\mathrm{SOMO}}\left(\overset{n}{\underset{t=p+1}{\prod }}T_{t}^{\left(1\right)}\right)$$ 70

Note that in the *p* = *q* case, there is not only one cut per orbital level, but also one additional cut around orbital *p* in Figure 9c. This allows us to write the central factor as the product *A*_*p*_^SOMO^ *B̃*_*p*_^(1), SOMO^ (see eq 70). In order to be able to use the same definitions of the factors, we had to introduce the restriction on which orbital levels can be used as indices for the intermediate spins (*S*_*t*–1_^′^ in eq 64 and *S*_*t*_ in eq 65).

The equations for the RMEs of the quintet double-excitation operators (Figures 11 and 12) after factorization are

$$p<q<r<s:\left\langle \Phi_{I}^{S^{\prime }}∥S_{pqrs}∥\Phi_{J}^{S}\right\rangle =\sqrt{15}\frac{1}{\sqrt{2S^{\prime }+1}}\zeta_{pq}\zeta_{rs}\times \left(\overset{p-1}{\underset{t=1}{\prod }}\delta_{S_{t},S_{t}^{\prime }}\right)B_{p}\left(\overset{q-1}{\underset{t=p+1}{\prod }}T_{t}^{\left(1/2\right)}\right){Ã}_{q}^{\left(1\right)}\left(\overset{r-1}{\underset{t=q+1}{\prod }}T_{t}^{\left(1\right)}\right)\times {\mathrm{B̃}}_{r}^{\left(3/2\right)}\left(\overset{s-1}{\underset{t=r+1}{\prod }}T_{t}^{\left(3/2\right)}\right){Ã}_{s}^{\left(2\right)}\left(\overset{n}{\underset{t=s+1}{\prod }}T_{t}^{\left(2\right)}\right)$$ 71

$$q<p<s<r:\left\langle \Phi_{I}^{S^{\prime }}∥S_{pqrs}∥\Phi_{J}^{S}\right\rangle =\sqrt{15}\frac{1}{\sqrt{2S^{\prime }+1}}\zeta_{pq}\zeta_{rs}\times \left(\overset{q-1}{\underset{t=1}{\prod }}\delta_{S_{t},S_{t}^{\prime }}\right)A_{q}\left(\overset{p-1}{\underset{t=q+1}{\prod }}T_{t}^{\left(1/2\right)}\right){\mathrm{B̃}}_{p}^{\left(1\right)}\left(\overset{s-1}{\underset{t=p+1}{\prod }}T_{t}^{\left(1\right)}\right)\times {Ã}_{s}^{\left(3/2\right)}\left(\overset{r-1}{\underset{t=s+1}{\prod }}T_{t}^{\left(3/2\right)}\right){\mathrm{B̃}}_{r}^{\left(2\right)}\left(\overset{n}{\underset{t=r+1}{\prod }}T_{t}^{\left(2\right)}\right)$$ 72

$$p<q<s<r:\left\langle \Phi_{I}^{S^{\prime }}∥S_{pqrs}∥\Phi_{J}^{S}\right\rangle =\sqrt{15}\frac{1}{\sqrt{2S^{\prime }+1}}\zeta_{pq}\zeta_{rs}\times \left(\overset{p-1}{\underset{t=1}{\prod }}\delta_{S_{t},S_{t}^{\prime }}\right)B_{p}\left(\overset{q-1}{\underset{t=p+1}{\prod }}T_{t}^{\left(1/2\right)}\right){Ã}_{q}^{\left(1\right)}\left(\overset{s-1}{\underset{t=q+1}{\prod }}T_{t}^{\left(1\right)}\right)\times {Ã}_{s}^{\left(3/2\right)}\left(\overset{r-1}{\underset{t=s+1}{\prod }}T_{t}^{\left(3/2\right)}\right){\mathrm{B̃}}_{r}^{\left(2\right)}\left(\overset{n}{\underset{t=r+1}{\prod }}T_{t}^{\left(2\right)}\right)$$ 73

$$q<p<r<s:\left\langle \Phi_{I}^{S^{\prime }}∥S_{pqrs}∥\Phi_{J}^{S}\right\rangle =\sqrt{15}\frac{1}{\sqrt{2S^{\prime }+1}}\zeta_{pq}\zeta_{rs}\times \left(\overset{q-1}{\underset{t=1}{\prod }}\delta_{S_{t},S_{t}^{\prime }}\right)A_{q}\left(\overset{p-1}{\underset{t=q+1}{\prod }}T_{t}^{\left(1/2\right)}\right){\mathrm{B̃}}_{p}^{\left(1\right)}\left(\overset{r-1}{\underset{t=p+1}{\prod }}T_{t}^{\left(1\right)}\right)\times {\mathrm{B̃}}_{r}^{\left(3/2\right)}\left(\overset{s-1}{\underset{t=r+1}{\prod }}T_{t}^{\left(3/2\right)}\right){Ã}_{s}^{\left(2\right)}\left(\overset{n}{\underset{t=s+1}{\prod }}T_{t}^{\left(2\right)}\right)$$ 74

$$p<r<q<s:\left\langle \Phi_{I}^{S^{\prime }}∥S_{pqrs}∥\Phi_{J}^{S}\right\rangle =\sqrt{15}\frac{1}{\sqrt{2S^{\prime }+1}}\zeta_{pq}\zeta_{rs}\times \left(\overset{p-1}{\underset{t=1}{\prod }}\delta_{S_{t},S_{t}^{\prime }}\right)B_{p}\left(\overset{r-1}{\underset{t=p+1}{\prod }}T_{t}^{\left(1/2\right)}\right){\mathrm{B̃}}_{r}^{\left(1\right)}\left(\overset{q-1}{\underset{t=r+1}{\prod }}T_{t}^{\left(1\right)}\right)\times {Ã}_{q}^{\left(3/2\right)}\left(\overset{s-1}{\underset{t=q+1}{\prod }}T_{t}^{\left(3/2\right)}\right){Ã}_{s}^{\left(2\right)}\left(\overset{n}{\underset{t=s+1}{\prod }}T_{t}^{\left(2\right)}\right)$$ 75

$$q<s<p<r:\left\langle \Phi_{I}^{S^{\prime }}∥S_{pqrs}∥\Phi_{J}^{S}\right\rangle =\sqrt{15}\frac{1}{\sqrt{2S^{\prime }+1}}\zeta_{pq}\zeta_{rs}\times \left(\overset{q-1}{\underset{t=1}{\prod }}\delta_{S_{t},S_{t}^{\prime }}\right)A_{q}\left(\overset{s-1}{\underset{t=q+1}{\prod }}T_{t}^{\left(1/2\right)}\right){Ã}_{s}^{\left(1\right)}\left(\overset{p-1}{\underset{t=s+1}{\prod }}T_{t}^{\left(1\right)}\right)\times {\mathrm{B̃}}_{p}^{\left(3/2\right)}\left(\overset{r-1}{\underset{t=p+1}{\prod }}T_{t}^{\left(3/2\right)}\right){\mathrm{B̃}}_{r}^{\left(2\right)}\left(\overset{n}{\underset{t=r+1}{\prod }}T_{t}^{\left(2\right)}\right)$$ 76

$$p<q=r<s:\left\langle \Phi_{I}^{S^{\prime }}∥S_{pqrs}∥\Phi_{J}^{S}\right\rangle =\sqrt{15}\frac{1}{\sqrt{2S^{\prime }+1}}\zeta_{pq}\zeta_{rs}\times \left(\overset{p-1}{\underset{t=1}{\prod }}\delta_{S_{t},S_{t}^{\prime }}\right)B_{p}\left(\overset{q-1}{\underset{t=p+1}{\prod }}T_{t}^{\left(1/2\right)}\right){Ã}_{q}^{\left(1\right),\mathrm{SOMO}}\times {\mathrm{B̃}}_{q}^{\left(3/2\right),\mathrm{SOMO}}\left(\overset{s-1}{\underset{t=q+1}{\prod }}T_{t}^{\left(3/2\right)}\right){Ã}_{s}^{\left(2\right)}\left(\overset{n}{\underset{t=s+1}{\prod }}T_{t}^{\left(2\right)}\right)$$ 77

$$q<p=s<r:\left\langle \Phi_{I}^{S^{\prime }}∥S_{pqrs}∥\Phi_{J}^{S}\right\rangle =\sqrt{15}\frac{1}{\sqrt{2S^{\prime }+1}}\zeta_{pq}\zeta_{rs}\times \left(\overset{q-1}{\underset{t=1}{\prod }}\delta_{S_{t},S_{t}^{\prime }}\right)A_{q}\left(\overset{p-1}{\underset{t=q+1}{\prod }}T_{t}^{\left(1/2\right)}\right){Ã}_{p}^{\left(1\right),\mathrm{SOMO}}\times {\mathrm{B̃}}_{p}^{\left(3/2\right),\mathrm{SOMO}}\left(\overset{r-1}{\underset{t=p+1}{\prod }}T_{t}^{\left(3/2\right)}\right){\mathrm{B̃}}_{r}^{\left(2\right)}\left(\overset{n}{\underset{t=r+1}{\prod }}T_{t}^{\left(2\right)}\right)$$ 78

$$p<q<r=s:\left\langle \Phi_{I}^{S^{\prime }}∥S_{pqrs}∥\Phi_{J}^{S}\right\rangle =\sqrt{15}\frac{1}{\sqrt{2S^{\prime }+1}}\zeta_{pq}\times \left(\overset{p-1}{\underset{t=1}{\prod }}\delta_{S_{t},S_{t}^{\prime }}\right)B_{p}\left(\overset{q-1}{\underset{t=p+1}{\prod }}T_{t}^{\left(1/2\right)}\right){Ã}_{q}^{\left(1\right)}\left(\overset{r-1}{\underset{t=q+1}{\prod }}T_{t}^{\left(1\right)}\right)\times {Ã}_{r}^{\left(3/2\right),\mathrm{SOMO}}{\mathrm{B̃}}_{r}^{\left(2\right),\mathrm{SOMO}}\left(\overset{n}{\underset{t=r+1}{\prod }}T_{t}^{\left(2\right)}\right)$$ 79

$$q<p<r=s:\left\langle \Phi_{I}^{S^{\prime }}∥S_{pqrs}∥\Phi_{J}^{S}\right\rangle =\sqrt{15}\frac{1}{\sqrt{2S^{\prime }+1}}\zeta_{pq}\times \left(\overset{q-1}{\underset{t=1}{\prod }}\delta_{S_{t},S_{t}^{\prime }}\right)A_{q}\left(\overset{p-1}{\underset{t=q+1}{\prod }}T_{t}^{\left(1/2\right)}\right){\mathrm{B̃}}_{p}^{\left(1\right)}\left(\overset{r-1}{\underset{t=p+1}{\prod }}T_{t}^{\left(1\right)}\right)\times {Ã}_{r}^{\left(3/2\right),\mathrm{SOMO}}{\mathrm{B̃}}_{r}^{\left(2\right),\mathrm{SOMO}}\left(\overset{n}{\underset{t=r+1}{\prod }}T_{t}^{\left(2\right)}\right)$$ 80

$$p=q<r<s:\left\langle \Phi_{I}^{S^{\prime }}∥S_{pqrs}∥\Phi_{J}^{S}\right\rangle =\sqrt{15}\frac{1}{\sqrt{2S^{\prime }+1}}\zeta_{rs}\times \left(\overset{p-1}{\underset{t=1}{\prod }}\delta_{S_{t},S_{t}^{\prime }}\right)A_{p}^{\mathrm{SOMO}}{\mathrm{B̃}}_{p}^{\left(1\right),\mathrm{SOMO}}\left(\overset{r-1}{\underset{t=p+1}{\prod }}T_{t}^{\left(1\right)}\right)\times {\mathrm{B̃}}_{r}^{\left(3/2\right)}\left(\overset{s-1}{\underset{t=r+1}{\prod }}T_{t}^{\left(3/2\right)}\right){Ã}_{s}^{\left(2\right)}\left(\overset{n}{\underset{t=s+1}{\prod }}T_{t}^{\left(2\right)}\right)$$ 81

$$p=q<s<r:\left\langle \Phi_{I}^{S^{\prime }}∥S_{pqrs}∥\Phi_{J}^{S}\right\rangle =\sqrt{15}\frac{1}{\sqrt{2S^{\prime }+1}}\zeta_{rs}\times \left(\overset{p-1}{\underset{t=1}{\prod }}\delta_{S_{t},S_{t}^{\prime }}\right)A_{p}^{\mathrm{SOMO}}{\mathrm{B̃}}_{p}^{\left(1\right),\mathrm{SOMO}}\left(\overset{s-1}{\underset{t=p+1}{\prod }}T_{t}^{\left(1\right)}\right)\times {Ã}_{s}^{\left(3/2\right)}\left(\overset{r-1}{\underset{t=s+1}{\prod }}T_{t}^{\left(3/2\right)}\right){\mathrm{B̃}}_{r}^{\left(2\right)}\left(\overset{n}{\underset{t=r+1}{\prod }}T_{t}^{\left(2\right)}\right)$$ 82

$$p=q<r=s:\left\langle \Phi_{I}^{S^{\prime }}∥S_{pqrs}∥\Phi_{J}^{S}\right\rangle =\sqrt{15}\frac{1}{\sqrt{2S^{\prime }+1}}\times \left(\overset{p-1}{\underset{t=1}{\prod }}\delta_{S_{t},S_{t}^{\prime }}\right)A_{p}^{\mathrm{SOMO}}{\mathrm{B̃}}_{p}^{\left(1\right),\mathrm{SOMO}}\left(\overset{r-1}{\underset{t=p+1}{\prod }}T_{t}^{\left(1\right)}\right)\times {Ã}_{r}^{\left(3/2\right),\mathrm{SOMO}}{\mathrm{B̃}}_{r}^{\left(2\right),\mathrm{SOMO}}\left(\overset{n}{\underset{t=r+1}{\prod }}T_{t}^{\left(2\right)}\right)$$ 83

#### 2.4.3 Comparison with Previous Work

At this point, a few words are in order on the relation of our equations with previous work. The early work by Drake, Kent, and Schlesinger employed a labeling of intermediate spins by electron indices.^43,55^ This leads to redudancies because e.g. the *T* and *M* factors, or the *B̃* and *C* factors of Kent and Schlesinger^55^ are (apart from details like the phases and square root prefactors) essentially just different manifestations of the same underlying structure. Furthermore, these authors always include two electron indices per factor, meaning either one DOMO or two SOMOs. Consequently, in the SOMO case all factors decompose into two factors, one of which is a *T*-type factor. In contrast, our definition of the factors always only involves a single orbital level. Incidentally, the *E* and *Ẽ* factors from Kent and Schlesinger^55^ turn out to be unnecessary when making this choice. We should also mention that our *A*_*t*_ factor can be better compared (apart from a sign and square root prefactor) with the *B* ^′^ factor of these authors than with their *A* factor.

Regarding earlier work applying graphical angular momentum coupling techniques to spin-dependent operators, our approach is most distinct in the treatment of the quintet double-excitation operator. Previous work led to more complicated expressions for the RMEs that even involved 9*j* symbols.^56–58^ In contrast, our treatment based on the sequentially coupled *S*_*ij*_^*m*^ operators (Figure 8) leads to much simpler expressions that only involve 6*j* symbols. Furthermore, the earlier treatments require the definition of a much larger number of different factors, while we demonstrated that all possible cases of RMEs for the triplet single-excitation and the quintet double-excitation operators can be compactly expressed in terms of only five types of factors (even only three if one introduces an additional spin 0 line as shown in Figure 14). We believe that this reduced complexity is very beneficial when it comes to the implementation of the equations.

#### 2.4.4 Determination of the Sign Factors

The equations derived in the previous Section 2.4.2 enable the calculation of matrix elements as a product of orbital-specific factors, apart from the signs ζ_*pq*_ and ζ_*pqrs*_. In this section we describe how the signs can also be implemented as a product of orbital-specific factors. The sign of a simple cyclic permutation *C*_*pq*_ is given by ζ_*pq*_ = (−1)^*i*_*p*_−*i*_*q*_^ (eq 30). For *C*_*pqrs*_ (see Table 1), one must distinguish two different cases: Those where the ranges of the permutations *C*_*pq*_ and *C*_*rs*_ are disjoint, and the two cases *p* < *r* < *q* < *s* and *q* < *s* < *p* < *r* where they overlap. In the nonoverlapping case, ζ_*pq*_ and ζ_*rs*_ can be considered separately. In the overlapping case it is helpful to define ζ̅_*pr*_ = (−1)^*i*_*p*_−*i*_*r*_^ and ζ̅_*qs*_ = (−1)^*i*_*q*_−*i*_*s*_^. With these definitions, one can write the overall sign as

$$\zeta_{pqrs}=\zeta_{pq}\zeta_{rs}=(-1{)}^{i_{p}-i_{q}}(-1{)}^{i_{r}-i_{s}}=(-1{)}^{i_{p}-i_{r}}(-1{)}^{i_{q}-i_{s}}={\mathrm{\zeta ̅}}_{pr}{\mathrm{\zeta ̅}}_{qs}$$ 84

This allows us (like in the nonoverlapping case) to consider separately the range from the first to the second, and from the third to the fourth orbital. Keeping in mind our choice that the electron indices *i*_*p*_, *i*_*q*_, *i*_*r*_, and *i*_*s*_ correspond to the right line in *A* or *Ã* factors and to the left line in *B* or *B̃* factors, one can derive the expressions for the overall sign for the range between the first and second orbital (out of *p*, *q*, *r*, and *s*) as shown in Table 2. One can observe that all cases have a (−1)^*n*_between_^ factor in common that can be implemented by assigning a minus sign to each *T*_*i*_^(1/2)^ factor, ζ­(*T* ^(1/2)^) = −1. With *n*_between_ we mean the number of SOMOs between the first and second orbital involved in the excitation. The remaining sign puts constraints on the other orbital-specific sign factors, which are also specified in Table 2. Similarly, for the orbital range between the third and fourth orbital, we can define ζ­(*T* ^(3/2)^) = −1 and obtain analogous constraints for the remaining factors. An overview of all constraints is shown in Figure 15. There are two possible choices for the factors that are compatible with these constraints. We choose the one that leads to fewer additional minus signs. Overall, this means that the signs ζ_*pq*_ (for the triplet single-excitation operator) and ζ_*pqrs*_ (for the quintet double-excitation operator) can be implemented by multiplying the factors *T*_*t*_^(1/2)^, *T*_*t*_^(3/2)^, *B*_*t*_^SOMO^, *Ã*_*t*_^(1), SOMO^, *B̃*_*t*_^(3/2), SOMO^, and *Ã*_*t*_^(2), SOMO^ (whenever they are occurring within the equation for an RME) with an additional minus sign.

**Table 2:** Complete Signs and Extraction of Constraints for Sign Factors for the First Orbital Range (the Second Orbital Range Works Completely Analogously)^a^

	nonoverlapping		overlapping	
case	*p* < *q*	*p* > *q*	ζ̅_*pr*_	ζ̅_*qs*_
complete sign	$\zeta_{pq}={\left(-1\right)}^{n_{\mathrm{between}}+\delta_{n_{p},2}+\delta_{n_{q},2}}$	$\zeta_{pq}={\left(-1\right)}^{n_{\mathrm{between}}}$	${\overset{-}{\zeta }}_{pr}=(-1{)}^{n_{\mathrm{between}}+\delta_{n_{p},2}+1}$	${\overset{-}{\zeta }}_{qs}=(-1{)}^{n_{\mathrm{between}}+\delta_{n_{s},2}+1}$
constraints	$$\begin{array}{cc} \zeta (B^{\mathrm{DOMO}}) & =\zeta ({\tilde{A}}^{(1),\mathrm{DOMO}})\\ \neq \zeta ({\tilde{A}}^{(1),\mathrm{SOMO}}) & =\zeta (B^{\mathrm{SOMO}}) \end{array}$$	$$\begin{array}{cc} \zeta ({\tilde{B}}^{(1),\mathrm{SOMO}}) & =\zeta ({\tilde{B}}^{(1),\mathrm{DOMO}})\\ =\zeta (A^{\mathrm{SOMO}}) & =\zeta (A^{\mathrm{DOMO}}) \end{array}$$	$$\begin{array}{cc} \zeta ({\tilde{B}}^{(1),\mathrm{SOMO}}) & =\zeta ({\tilde{B}}^{(1),\mathrm{DOMO}})\\ =\zeta (B^{\mathrm{DOMO}}) & \neq \zeta (B^{\mathrm{SOMO}}) \end{array}$$	$$\begin{array}{cc} \zeta ({\tilde{A}}^{(1),\mathrm{SOMO}}) & \neq \zeta ({\tilde{A}}^{(1),\mathrm{DOMO}})\\ =\zeta (A^{\mathrm{SOMO}}) & =\zeta (A^{\mathrm{DOMO}}) \end{array}$$

a *n*_*p*_ is the occupation number of orbital *p*, i.e., the sign ${\left(-1\right)}^{\delta_{n_{p},2}}$ occurs if *p* is a DOMO.

**Figure 15:**
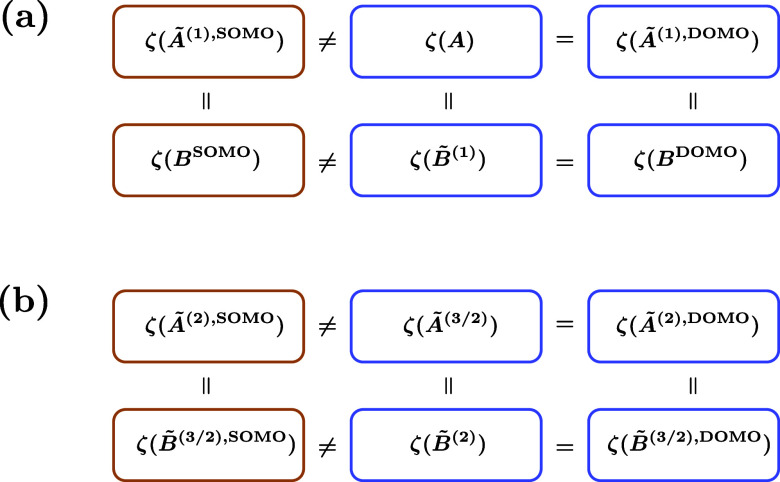
Visualization of the constraints for orbital-specific sign factors given in Table 2. (a) gives the constraints for the range between the first and second, and (b) the constraints for the range between the third and fourth orbital (out of *p*, *q*, *r*, and *s*). The two colors visualize the two different signs (plus and minus). In this work, we choose “minus” for the sign factors in light brown (left column) and “plus” for the remaining ones.

## 3 Implementation

The following was implemented in a development version of ORCA^59,60^ and will become publicly available with the next release.

### 3.1 Calculation of Matrix Elements of Triplet Operators for ICE States

We start by combining eqs 5 and 7, which gives

$$\left\langle \Psi_{I}^{S^{\prime }}∥A_{T}\left(m^{\prime }\right)∥\Psi_{J}^{S}\right\rangle =\underset{KL}{\sum }C_{KI}^{S^{\prime }}C_{LJ}^{S}\underset{pq}{\sum }a_{pq}\left(m^{\prime }\right)\left\langle \Phi_{K}^{S^{\prime }}∥s_{pq}∥\Phi_{L}^{S}\right\rangle$$ 85

Most pairs of CSFs are not connected by a single excitation, i.e., there is sparsity that can be exploited. For a given bra CSF with label *K*, we define S­(*K*) (not to be confused with the invariance group defined in Section 2.2) as the set of triples (*L*, *p*, *q*) such that the corresponding coupling coefficient RMEs ⟨Φ_*K*_^*S* ^′^^∥*s*_*pq*_∥Φ_*L*_^*S*^⟩ are nonzero. This allows us to rewrite eq 85 as

$$\left\langle \Psi_{I}^{S^{\prime }}∥A_{T}\left(m^{\prime }\right)∥\Psi_{J}^{S}\right\rangle =\underset{K}{\sum }C_{KI}^{S^{\prime }}\underset{L,p,q\in S\left(K\right)}{\sum }C_{LJ}^{S}a_{pq}\left(m^{\prime }\right)\left\langle \Phi_{K}^{S^{\prime }}∥s_{pq}∥\Phi_{L}^{S}\right\rangle$$ 86

In order to evaluate eq 86 efficiently, it is convenient to define an intermediate quantity

$$V_{KJ}^{S^{\prime }S}\left(m^{\prime }\right)=\underset{L,p,q\in S\left(K\right)}{\sum }C_{LJ}^{S}a_{pq}\left(m^{\prime }\right)\left\langle \Phi_{K}^{S^{\prime }}∥s_{pq}∥\Phi_{L}^{S}\right\rangle$$ 87

Eq 86 leads to the algorithm shown in Figure 16, which is evaluated separately for each unique pair of spin quantum numbers *S* ^′^ and *S* of the bra and ket CSFs. Since the loop iterations over the bra CSFs *K* are independent, the algorithm can be easily parallelized by calculating the contribution from different bra CSFs on different CPU cores.

**Figure 16:**
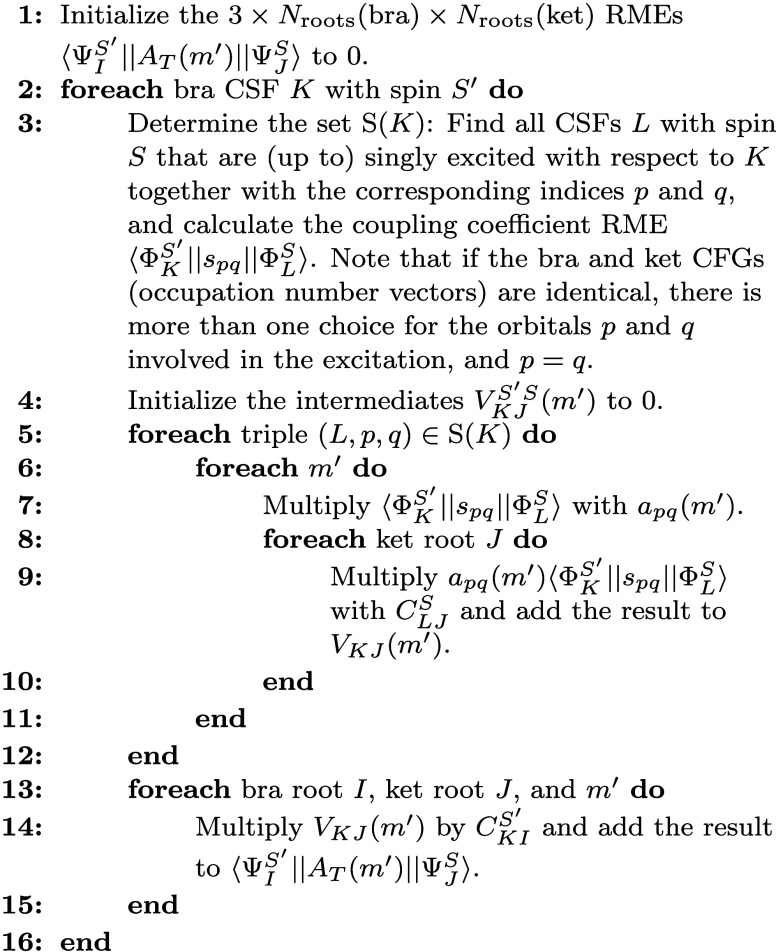
Pseudocode for the calculation of the RMEs of a triplet one-electron operator.

The only important part of this algorithm that was not yet explained in detail is the determination of the set *S*(*K*) for a given bra CSF *K* and the corresponding coupling coefficient RMEs ⟨Φ_*K*_^*S* ^′^^∥*s*_*pq*_∥Φ_*L*_^*S*^⟩. This part will be explained in the following section.

### 3.2 Calculation of Coupling Coefficient RMEs for the Triplet Single-Excitation Operator

In the ICE method, individual CSFs are represented as sequences containing one of the four symbols 0, 1^+^, 1^–^, 2 for each of the active orbitals. 0 and 2 represent VMOs and DOMOs, respectively, and 1^+^, 1^–^ represent SOMOs for which the intermediate spin is increased or decreased by 1/2, respectively. Sets of CSFs, e.g. all selected CSFs for a given spin multiplicity, are stored in a tree data structure. Each node (including the root node) can have up to four children corresponding to the four different symbols 0, 1^+^, 1^–^, 2. Each CSF is then in one-to-one correspondence with a path through the tree from the root node to one of the leaf nodes, where each node (except the root node) corresponds to one active orbital. Note that trailing zeros are not explicitly stored in the tree (since they can be implied from the fixed total electron number).

The algorithm presented in the previous section requires a function that can, given an arbitrary bra CSF *K* and a tree representing all possible CSFs occurring in the ket, generate the set S­(*K*). This is realized in our implementation by a recursive function called FindSingles_triplet_OTF (where OTF stands for “on the fly”) that walks the tree from root node to leaf nodes and accumulates the coupling coefficients on the fly. The idea to evaluate coupling coefficients on the fly was pioneered in the FindSingles_OTF function used in the spin-independent ICE method to calculate *E*_*pq*_ coupling coefficients.^23,24^ The function takes as arguments a node of the tree, the current orbital level (an integer number), the total number of occupation number differences between bra and ket up to this point (Δ*n*_occ_), the accumulated total bra and ket spins and electron numbers, the indices *p* and *q* of the orbitals involved in the excitation (if they were already found; they are initialized to –1), as well as the accumulated coupling coefficient RME up to this point. Then, for all child nodes of the present node (up to four), the corresponding factors belonging to the next orbital level are constructed and multiplied with the previous accumulated coupling coefficient RME. Afterward, FindSingles_triplet_OTF is called again with the respective child node and the updated coupling coefficient RME as arguments. As soon as a leaf node is reached, the accumulated coupling coefficient RME is stored together with other important information like the orbitals *p*, *q* involved in the excitation. The recursion is started by calling the function with the root node as its argument, and the tree is traversed in preorder traversal. An advantage of this recursive approach is that there is no redundant work. For example, consider (an artificial example of) a tree that has uniformly the same depth *n* and same number of child nodes *c* everywhere. Then there are *c* ^*i*^ nodes corresponding to orbital level *i*. The total number of nodes in the tree is

$$\overset{n}{\underset{i=0}{\sum }}c^{i}=\frac{c^{n+1}-1}{c-1}$$ 88

whereas the total number of CSFs times the total number of orbital levels (which corresponds to the naive number of factors to evaluate) is given by ${nc}^{n}$ . Considering the example *c* = 4 and *n* = 20, this corresponds to about 15 times more evaluated factors in the “naive” implementation, i.e., on average a 15-fold redundancy.

Note that for each of the three types of factors (*A*, *B*, *T*), one needs to distinguish four cases, depending on whether the spin on the operator line is 1/2, 1, 3/2, or 2. This information is accessed in the FindSingles_triplet_OTF function via the variable Δ*n*_occ_. The structure of the function is shown in Figure 17. Note that the trivial factor “1” occurs in the following cases: If the occupation number is 2 in both bra and ket, if it is 0 in both bra and ket, or if it is 1 in both bra and ket and Δ*n*_occ_ = 0. Also note that in cases where the next bra occupation number and the next ket occupation number are both 1, we call FindSingles_triplet_OTF a second time to account for the case *p* = *q*.

**Figure 17:**
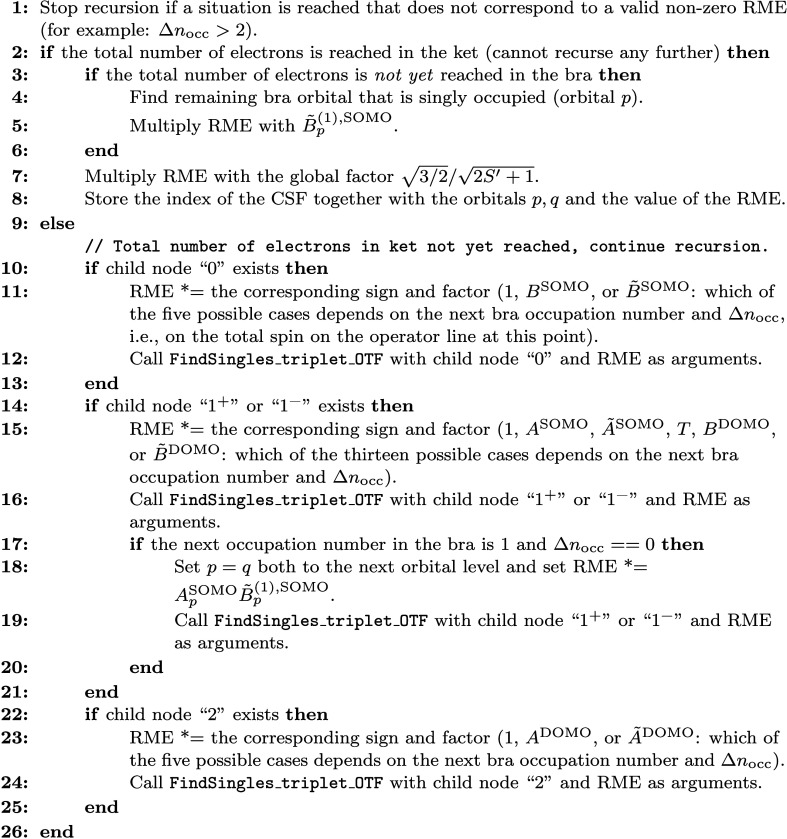
Pseudocode of the FindSingles_triplet_OTF function for calculating the triplet coupling coefficient RMEs.

### 3.3 Implementation of the Direct Spin–Spin Coupling Operator

Employing again sparsity, i.e., that most pairs of CSFs do not have nonzero matrix elements of quintet double excitation operators, eq 16 becomes

$$\left\langle \Psi_{I}^{S^{\prime }}∥H_{\mathrm{SSC}}\left(m^{\prime }\right)∥\Psi_{J}^{S}\right\rangle =\underset{K}{\sum }C_{KI}^{S^{\prime }}\underset{L,p,q,r,s\in S\left(K\right)}{\sum }C_{LJ}^{S}{\left(-1\right)}^{m^{\prime }}\left[d_{pqrs}^{\left(-m^{\prime }\right)}-d_{psrq}^{\left(-m^{\prime }\right)}\right]\times \left\langle \Phi_{K}^{S^{\prime }}∥S_{pqrs}∥\Phi_{L}^{S}\right\rangle$$ 89

Here, for a given bra CSF *K*, S­(*K*) denotes the sets of quintuples (*L*, *p*, *q*, *r*, *s*) (with the restriction *p* < *r*, *q* < *s*) such that ⟨Φ_*K*_^*S* ^′^^∥*S*_*pqrs*_∥Φ_*L*_^*S*^⟩ is nonzero. Eq. 90 can be implemented in complete analogy to the triplet operator case (Figures 16 and 17). In order to determine the sets S­(*K*) and the corresponding coupling coefficient RMEs, we implemented a function FindDoubles_quintet_OTF. The only important differences to the FindSingles_triplet_OTF function are the stopping conditions (e.g., Δ*n*_occ_ > 4), the fact that now four orbitals *p*, *q*, *r*, *s* involved in the excitation are stored, that both *p*, *r* might still be undetermined when the total electron number in the ket is reached, and that the RME is multiplied with a global prefactor of $\sqrt{15}/\sqrt{2S^{\prime }+1}$ instead of $\sqrt{3/2}/\sqrt{2S^{\prime }+1}$ . Furthermore, the additional branching for the case where an electron is annihilated and created in the same orbital is always done if Δ*n*_occ_ ≤ 2 (not only for Δ*n*_occ_ = 0).

## 4 Illustrative Applications

In this section, we illustrate the capabilities of our new implementation with two examples: The calculation of *g*-values in a Mo^III^ complex also studied by Roemelt^30^ and the ZFS in dioxygen. We only sketch the computational details in the following and refer to the ORCA input files provided by us for more details.

The first example is a truncated model complex for the [HIPTN_3_N]­MoNH_3_ complex reported by McNaughton et al.,^61^ which is an intermediate in the catalytic reduction of N_2_ to NH_3_. It is a pseudotrigonal low-spin *d* ^3^ complex (*S* = 1/2) with a formal ^2^E ground state that is split into two Kramers doublets by Jahn–Teller distortion and SOC. Its electron paramagnetic resonance g-factors were not only measured but also calculated by Roemelt^30^ using the DMRG/QDPT approach. We tried to follow as closely as possible the computational protocol of Roemelt, who performed a DMRG­(49,33) calculation with an active space consisting of the Mo 3*s*, 3*d*, 4*d*, 5*d* orbitals, all 2*s* and 2*p* orbitals of the coordinating N atoms of the [HIPTN_3_N]^3–^ ligand, and the 2*p*_*z*_ orbital on the ammonia ligand. We started with model 1” of McNaughton et al.^61^ with an NH_3_ ligand (see Figure 18), obtained orbitals of the Mo^III^ ion and the ligands separately, and combined them with the orca_mergefrag program. These orbitals were used as starting guess for a CASSCF­(3,5) with the metal 4*d* orbitals as active orbitals, using DKH2 scalar relativity, the SARC-DKH-TZVP basis set for Mo and the DKH2-def2-TZVP for all other atoms, and localizing the internal, active, and external orbitals separately using the Pipek–Mezey^62^ method. After localization, only 2*s* and 2*p* orbitals on the ligands could be readily identified, whereas some of the Mo-based orbitals were heavily mixed. Therefore, the blocks 0–17 (Mo core orbitals), 88–92 (active Mo 4*d* orbitals), and 93–146 (virtual orbitals localized on Mo) of the Fock matrix in the localized orbital basis were separately diagonalized with the orca_blockf program in order to obtain block-canonicalized orbitals. After the block-canonicalization, the Mo 3*s* and 3*d* orbitals in the core, and the Mo 5*d* orbitals in the virtual space, could be readily identified. Using these orbitals, we finally set up an ICE­(49,33) calculation with two doublet roots followed by a QDPT^44^ calculation incorporating the effect of SOC. The g-factors obtained with the equation described by Gerloch and McMeeking^63^ and Bolvin^64^ are 1.29, 1.29, 3.41, which is relatively similar to the *g*-factors 1.30, 1.30, 3.34 obtained by Roemelt. There is still a sizable difference of both results from the experimental g-factors of 1.35, 1.35, 3.20.^61^ In comparison, a standard small active space CASSCF/QDPT calculation with 3 electrons in 5 active metal *d*-orbitals yields 1.33, 1.33, 3.34. These results are even closer to the experiment than our ICE numbers or Roemelt’s DMRG numbers. Correcting diagonal energies by NEVPT2 calculations in order to incorporate dynamic correlation effects – which in principle should improve the theoretical description – leads to *g*-factors of 1.19, 1.19, 3.43, which are significantly further from the experimental numbers. This is a hint that the excellent agreement at the CASSCF/QDPT level with a small active space might benefit from a fortuitous cancellation of errors. The remaining discrepancy at the ICE/QDPT level is also not surprising, given that we used a truncated model complex and neglected dynamic electron correlation, vibrational and dynamical effects, and solvent effects. However, the purpose here is not to obtain extremely accurate agreement with the experiment, but to highlight the size of active spaces for which we can now calculate the effects of spin-dependent operators.

**Figure 18:**
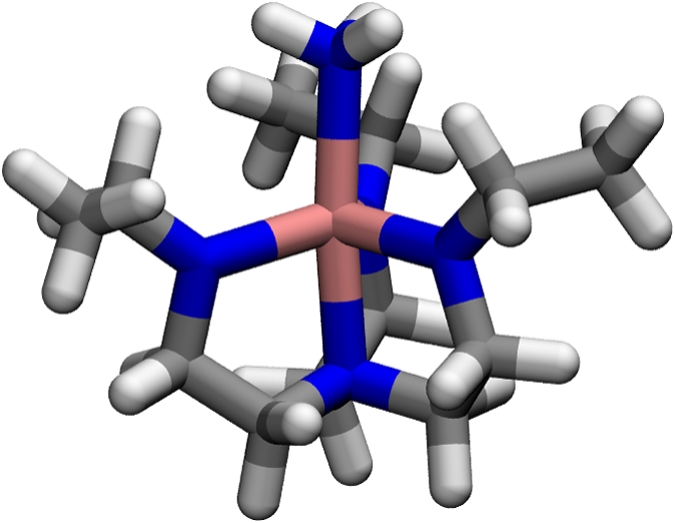
Truncated model complex for [HIPTN_3_N]­MoNH_3_.

The second investigated example is the ZFS in dioxygen. We assumed a bond length of 1.2058 Å (the structure obtained at the CCSD­(T) level with the cc-pCVQZ basis set and all orbitals correlated as deposited in the NIST Computational Chemistry Comparison and Benchmark Database (CCCBDB)^65^) and employed the def2-TZVP basis set. Initially, we performed a state-averaged CASSCF­(2,2) calculation including the lowest triplet and the lowest three singlet roots. These are the well-known low-energy states of the molecule that originate from distributing two electrons over the two π* orbitals. Based on these orbitals, we then performed ICE­(12,60) calculations, which corresponds to approximate FCI in the def2-TZVP basis set except for the oxygen 1*s* orbitals, which are not correlated. We compared two different ICE­(12,60) calculations, one in which only the lowest triplet and the lowest 3 singlet roots were included, and one where the lowest 5 triplet roots and the lowest 4 singlet roots were included, in order to investigate the convergence of the ZFS with increasing the size of the QDPT space. The states of the smaller QDPT space are those that result from distributing 2 electrons over the 2 valence π* orbitals. In the larger QDPT space, we added states up to ≈9 eV above the ground state originating from a single excitation from the π to the π* orbitals. The nonrelativistic energies are shown together with the experimentally fitted potential energy curves of O_2_ in Figure 19. The ZFS is 3.925 cm^–1^ with the smaller number of roots and 3.929 cm^–1^ with the larger number of roots, which shows that the value is essentially converged after including only the ground state and the lowest three singlet roots. It is well-known that SSC is crucial for describing the ZFS in dioxygen, and indeed, without it we obtain values of only 2.385 and 2.390 cm^–1^, respectively. The experimental value is 3.96 cm^–1^,^66^ which is quite close to the calculated value. The remaining error is probably mostly due to the approximate nature of the relativistic Hamiltonian used (in particular the spin–orbit mean field approximation) and the basis set error. In comparison, a standard small active space CASSCF/QDPT calculation with 6 electrons in 4 active orbitals (π and π*) yields a splitting of 3.096 cm^–1^, which agrees much less with the experimental value than our ICE/QDPT calculations. Adding dynamic correlation by replacing diagonal energies with NEVPT2 energies gives an even worse splitting of 2.481 cm^–1^ because of increased excitation energies. The excellent performance of our ICE/QDPT approach is due to the treatment of dynamic correlation effects in this small molecule, where the “active” space comprises all orbitals except for the oxygen 1*s* core orbitals. Note that our implementation features all spin-allowed Hamiltonian blocks of the SSC operator (i.e., Δ*S* = 0, ± 1, ± 2), whereas earlier implementations of SSC in the ORCA MRCI and CASSCF modules only considered the diagonal *S* = *S* ^′^ block. We added the possibility to only consider this block also in our ICE implementation and found that the off-diagonal blocks play a negligible role at least for the example of the O_2_ molecule.

**Figure 19:**
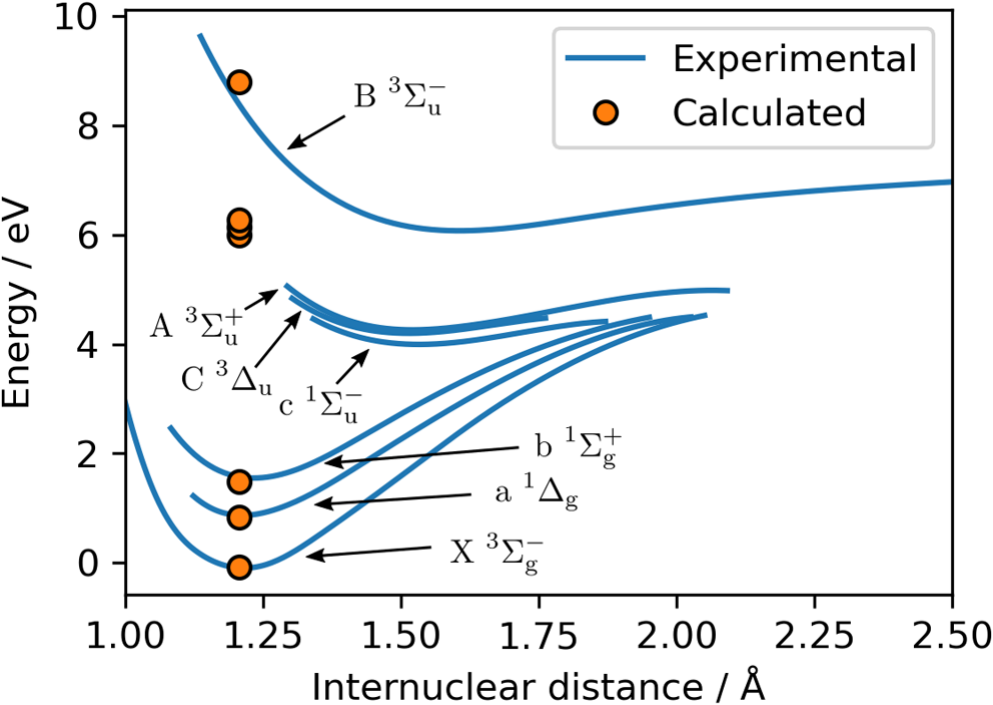
Experimentally fitted potential energy curves for the lowest electronic states of dioxygen and the corresponding calculated nonrelativistic energies at the minimum bond distance of the ground state. Note that only a single averaged energy is plotted for the orbitally degenerate states. The experimental curves were obtained by digitizing Figure 2 from Krupenie^67^ and smoothing the resulting data via a cubic spline fit.

We also checked the correctness of our implementation by comparing the ZFS in O_2_ with the already existing implementation in the ORCA MRCI module for a relatively small active space of 8 electrons in 10 orbitals. In this case, the MRCI module was run without excitations (i.e., performing just a CASCI) and setting the thresholds Tsel and Tpre to exactly 0.0. The ICE calculation was run with the thresholds Tgen and Tvar set to 0.0 and neglecting the coupling of different multiplicities via the SSC operator. Setting the thresholds to zero has in both cases the effect that the CI space is not truncated. When only considering SOC, we obtain a value of 2.202 cm^–1^ with both methods and when additionally considering SSC, we obtain a value of 3.766 cm^–1^ with the MRCI method and 3.767 cm^–1^ with the ICE method. Those results are for all practical purposes identical and demonstrate the correctness of our implementation.

Finally, we comment on the computational time needed for the QDPT calculation, using O_2_ with the larger number of 9 roots as an example. This calculation was performed on a machine with 24 Intel Xeon E5-2687W v4 CPU cores. In this case, the converged CI space contained about 9.66 million CSFs for the triplet block and about 6 million CSFs for the singlet block. Each nonrelativistic CI iteration (dominated by the σ vector construction, i.e., multiplication of the trial CI vector by the Hamiltonian matrix) took on average 13.8 and 5.9 h, respectively, for those two multiplicity blocks. In contrast, the calculation of the SOC RMEs took 1.6 h and the calculation of the SSC RMEs 31.4 h. Considering that the SSC RMEs also include the coupling of the two multiplicity blocks (which is not the case for the σ vector construction), the calculation of the SSC RMEs is roughly as efficient as the nonrelativistic code. The total computational time for the nonrelativistic CI phase, including multiple ICE macro-iterations with CI spaces increasing in size and between 6 and 15 CI iterations until convergence, was over 19 days. Overall, the QDPT calculation was responsible for 6.1% of the complete runtime, which is a small overhead on top of the nonrelativistic ICE calculation.

## 5 Conclusions

We presented an implementation of one-electron triplet operators (like effective one-electron spin–orbit coupling) and two-electron quintet operators (direct electronic spin–spin coupling) for use in QDPT calculations on top of nonrelativistic/scalar-relativistic ICE wave functions expressed in a CSF basis.

At the core of our method is the calculation of coupling coefficient RMEs as products of orbital-specific factors, similar to the factorization into segment factors in GUGA. We obtained the working equations by generalizing a result by Wormer and Paldus that expresses matrix elements of excitation operators between CSFs in terms of matrix elements of pure spin functions, and by using the graphical techniques of angular momentum analysis. This approach allowed us to derive working equations for the SSC operator that are much simpler than anything else published until now, and make use of the same types of factors that are already needed for the one-electron SOC operator.

We illustrated the capabilities of our implementation by two examples: The EPR *g*-factors of a Mo^III^-based catalytic intermediate, employing an active space of 49 electrons in 33 orbitals, and the ZFS tensor in dioxygen, with an active space of 12 electrons in 60 orbitals, which, apart from the frozen oxygen 1*s* orbitals, corresponds to a FCI calculation in the chosen triple-ζ basis.

We believe that the new possibility to perform QDPT calculations in ORCA based on ICE wave functions opens up many opportunities for exciting applications. One drawback of the approach is that it is sensitive to the number of nonrelativistic/scalar-relativistic excited states included into the QDPT procedure, and that potentially large numbers of states are needed for converged results. This is in particular true for polynuclear metal clusters, for which a better approach would be a one-step spin–orbit CI. We intend to explore this possibility within the ICE framework in future work.

## Data Availability

The data underlying this study are openly available on Zenodo at 10.5281/zenodo.15421594. The data extraction from ORCA output files was performed with a Snakemake^68^ workflow that can be obtained from https://github.com/LucasLang/ICE_SOC_SSC_analysis and 10.5281/zenodo.15421587.

## Appendix

### A.1 Proof that Any CSF Is Identical to Eq 17 with a Geminally Antisymmetric Spin Function

Without loss of generality, one can assume that the orbitals are ordered such that all SOMOs come before all DOMOs in the orbital string. Given a specific set of orbital occupation numbers (or equivalently: a specific Hartree product), one can write down any possible Slater determinant having these occupation numbers via

$$|\Phi_{\sigma_{1}\cdots \sigma_{n_{\mathrm{SOMOs}}}}\rangle =\sqrt{N!}A\left[\left(\overset{n_{\mathrm{SOMOs}}}{\underset{p=1}{\prod }}\psi_{p}\sigma_{p}\right)\left(\overset{n_{\mathrm{SOMOs}}+n_{\mathrm{DOMOs}}}{\underset{p=n_{\mathrm{SOMOs}}+1}{\prod }}\psi_{p}{\alpha \psi }_{p}\beta \right)\right]=\sqrt{N!}A\left[\left(\overset{n_{\mathrm{SOMOs}}}{\underset{p=1}{\prod }}\psi_{p}\sigma_{p}\right)\left(\overset{n_{\mathrm{SOMOs}}+n_{\mathrm{DOMOs}}}{\underset{p=n_{\mathrm{SOMOs}}+1}{\prod }}\psi_{p}\psi_{p}\frac{1}{2}\left(\alpha \beta -\beta \alpha \right)\right)\right]$$
90

Here, the σ_*p*_ are the spins (α or β) of the SOMOs. A normalized CSF is a linear combination of these Slater determinants and can be written as

$$\left|\Phi_{I}^{SM}\rangle =\underset{\sigma_{1}\cdots \sigma_{n_{\mathrm{SOMOs}}}\in \left\{\alpha ,\beta \right\}}{\sum }C_{\sigma_{1}\cdots \sigma_{n_{\mathrm{SOMOs}}}}\right|\Phi_{\sigma_{1}\cdots \sigma_{n_{\mathrm{SOMOs}}}}\rangle =\sqrt{N!}{\left(\frac{1}{\sqrt{2}}\right)}^{n_{\mathrm{DOMOs}}}A\left[|\Phi_{I}\rangle |X_{I}^{SM}\rangle \right]$$
91

with

$$|\Phi_{I}\rangle =\left(\overset{n_{\mathrm{SOMOs}}}{\underset{p=1}{\prod }}\psi_{p}\right)\left(\overset{n_{\mathrm{SOMOs}}+n_{\mathrm{DOMOs}}}{\underset{p=n_{\mathrm{SOMOs}}+1}{\prod }}\psi_{p}\psi_{p}\right)$$
92

$$|X_{I}^{SM}\rangle =\underset{\sigma_{1}\cdot \cdot \cdot \sigma_{n_{\mathrm{SOMOs}}}\in \left\{\alpha ,\beta \right\}}{\sum }C_{\sigma_{1}\cdot \cdot \cdot \sigma_{n_{\mathrm{SOMOs}}}}\left(\overset{n_{\mathrm{SOMOs}}}{\underset{p=1}{\prod }}\sigma_{p}\right)\times \left(\overset{n_{\mathrm{SOMOs}}+n_{\mathrm{DOMOs}}}{\underset{p=n_{\mathrm{SOMOs}}+1}{\prod }}\frac{1}{\sqrt{2}}\left(\alpha \beta -\beta \alpha \right)\right)$$
93

The spin function eq 94 is obviously geminally antisymmetric and normalized and eq 92 is identical to eq 17.

### A.2 A Rederivation of the Wigner–Eckart Theorem

Let *T*_*q*_^(*k*)^ be the *q*-component of a spin-*k* spin tensor operator. We first define the coupled states

$$\left|{\left(T^{\left(k\right)}X_{J}\right)}^{S^{\prime }M^{\prime }}\rangle =\underset{qM}{\sum }\left(\begin{array}{cc} k & S\\ q & M \end{array}|\begin{array}{c} S^{\prime }\\ M^{\prime } \end{array}\right)T_{q}^{\left(k\right)}\right|X_{J}^{SM}\rangle$$
94

It is straightforward to show that these states (if they are not vanishing) indeed have total spin *S* ^′^ and magnetic quantum number *M* ^′^. Since the operation in eq 95 is invertible, one can write

$$T_{q}^{\left(k\right)}\left|X_{J}^{SM}\rangle =\underset{S^{\prime }M^{\prime }}{\sum }\left(\begin{array}{cc} k & S\\ q & M \end{array}|\begin{array}{c} S^{\prime }\\ M^{\prime } \end{array}\right)\right|{\left(T^{\left(k\right)}X_{J}\right)}^{S^{\prime }M^{\prime }}\rangle$$
95

where we used the orthogonality of the CGCs,

$$\underset{S^{\prime }M^{\prime }}{\sum }\left(\begin{array}{cc} k & S\\ \mathrm{q̃} & \mathrm{M̃} \end{array}|\begin{array}{c} S^{\prime }\\ M^{\prime } \end{array}\right)\left(\begin{array}{cc} k & S\\ q & M \end{array}|\begin{array}{c} S^{\prime }\\ M^{\prime } \end{array}\right)=\delta_{q,\begin{array}{c} \mathrm{q̃} \end{array}}\delta_{M,\begin{array}{c} \mathrm{M̃} \end{array}}$$
96

Projecting on eq 96 from the left with another state having spin quantum numbers *S* ^′^ and *M* ^′^ gives

$$\left\langle X_{I}^{S^{\prime }M^{\prime }}\right|T_{q}^{\left(k\right)}|X_{J}^{SM}\rangle =\left(\begin{array}{cc} k & S\\ q & M \end{array}|\begin{array}{c} S^{\prime }\\ M^{\prime } \end{array}\right)\left\langle X_{I}^{S^{\prime }M^{\prime }}\right|{\left(T^{\left(k\right)}X_{J}\right)}^{S^{\prime }M^{\prime }}\rangle =\frac{1}{2S^{\prime }+1}\left(\begin{array}{cc} k & S\\ q & M \end{array}|\begin{array}{c} S^{\prime }\\ M^{\prime } \end{array}\right)\underset{M^{\prime }}{\sum }\left\langle X_{I}^{S^{\prime }M^{\prime }}\right|{\left(T^{\left(k\right)}X_{J}\right)}^{S^{\prime }M^{\prime }}\rangle$$
97

In the second step, we used that the inner product of two states with the same spin quantum numbers is *independent* of the magnetic quantum number. One can now insert the definition eq 95 into eq 98 and switch the order of the coupled spins in the CGCs to obtain the Wigner–Eckart theorem in the form

$$\left\langle X_{I}^{S^{\prime }M^{\prime }}\right|T_{q}^{\left(k\right)}|X_{J}^{SM}\rangle =\left(\begin{array}{cc} S & k\\ M & q \end{array}|\begin{array}{c} S^{\prime }\\ M^{\prime } \end{array}\right)\left\langle X_{I}^{S^{\prime }}∥T^{\left(k\right)}∥X_{J}^{S}\right\rangle$$
98

with the reduced matrix element (RME) given by

$$\left\langle X_{I}^{S^{\prime }}∥T^{\left(k\right)}∥X_{J}^{S}\right\rangle =\frac{1}{2S^{\prime }+1}\underset{qMM^{\prime }}{\sum }\left(\begin{array}{cc} S & k\\ M & q \end{array}|\begin{array}{c} S^{\prime }\\ M^{\prime } \end{array}\right)\left\langle X_{I}^{S^{\prime }M^{\prime }}\right|T_{q}^{\left(k\right)}|X_{J}^{SM}\rangle$$
99

## †

Technische Universität Berlin, Institut für Chemie, Theoretische Chemie/Quantenchemie, 10623 Berlin, Germany

## ‡

Aix Marseille Univ, CNRS, Centrale Méditerranée, iSm2, 13013 Marseille, France.
